# Learning Deep Representations of Cardiac Structures for 4D Cine MRI Image Segmentation through Semi-Supervised Learning

**DOI:** 10.3390/app122312163

**Published:** 2022-11-28

**Authors:** S. M. Kamrul Hasan, Cristian A. Linte

**Affiliations:** 1 Center for Imaging Science, Rochester Institute of Technology, Rochester, NY 14623, USA; 2 Department of Biomedical Engineering, Rochester Institute of Technology, Rochester, NY 14623, USA

**Keywords:** augmentation, cardiac segmentation, domain invariant features, disentangled representation, generative adversarial network, image quality, mutual information, reconstruction, variational autoencoder

## Abstract

Learning good data representations for medical imaging tasks ensures the preservation of relevant information and the removal of irrelevant information from the data to improve the interpretability of the learned features. In this paper, we propose a semi-supervised model—namely, combine-all in semi-supervised learning (C*q*SL)—to demonstrate the power of a simple combination of a disentanglement block, variational autoencoder (VAE), generative adversarial network (GAN), and a conditioning layer-based reconstructor for performing two important tasks in medical imaging: segmentation and reconstruction. Our work is motivated by the recent progress in image segmentation using semi-supervised learning (SSL), which has shown good results with limited labeled data and large amounts of unlabeled data. A disentanglement block decomposes an input image into a domain-invariant spatial factor and a domain-specific non-spatial factor. We assume that medical images acquired using multiple scanners (different domain information) share a common spatial space but differ in non-spatial space (intensities, contrast, etc.). Hence, we utilize our spatial information to generate segmentation masks from unlabeled datasets using a generative adversarial network (GAN). Finally, to reconstruct the original image, our conditioning layer-based reconstruction block recombines spatial information with random non-spatial information sampled from the generative models. Our ablation study demonstrates the benefits of disentanglement in holding domain-invariant (spatial) as well as domain-specific (non-spatial) information with high accuracy. We further apply a structured $L_{2}$ similarity $\left(SL_{2}\text{SIM}\right)$ loss along with a mutual information minimizer (MIM) to improve the adversarially trained generative models for better reconstruction. Experimental results achieved on the STACOM 2017 ACDC cine cardiac magnetic resonance (MR) dataset suggest that our proposed (C*q*SL) model outperforms fully supervised and semi-supervised models, achieving an 83.2% performance accuracy even when using only 1% labeled data. We hypothesize that our proposed model has the potential to become an efficient semantic segmentation tool that may be used for domain adaptation in data-limited medical imaging scenarios, where annotations are expensive. Code, and experimental configurations will be made available publicly.

## 1. Introduction

### 1.1. Background and Problem Statement

The emerging success of deep convolutional neural networks (CNNs) has rendered them the de facto model in solving high-level computer vision tasks [1–3]. However, such approaches mostly rely on large amounts of annotated data for training, the acquisition of which is expensive and laborious, especially for medical imaging/diagnostic radiology data. To address the need for high performance, there has been a growing trend in using a limited amount of annotated data along with an abundance of unlabeled data in a semi-supervised learning (SSL) setting.

The recent dominant body of research that has proposed SSL methods in deep learning features various approaches, including an auxiliary loss term defined on un-annotated data (consistency regularization) [4,5], adversarial networks [6], generating pseudo-labels [7,8] based on model predictions on weakly augmented unannotated data, self-training [9,10], adversarial learning [11] and domain adaptation [12]. Here we acknowledge their latest accomplishments in the field of domain adaptation, semi-supervised learning and interpretable representation learning by disentanglement and briefly discuss some of their yet outstanding limitations.

### 1.2. Ongoing Efforts and Related Work

#### Semi-Supervised Learning:

Semi-supervised learning (SSL) [13,14] has experienced much research attention thanks to the increasing availability of large-scale unlabeled data. Semi-supervised learning aims to revamp the model performance by learning from a small portion of labeled data along with optimizing an additional unsupervised loss on a larger portion of unlabeled data, assumed to be sampled from similar distributions, depending on the type of information that needs to be captured from the unlabeled data. Commonly, the rationale of SSL is based on generative models and adversarial networks. The integration of consistency regularization in SSL has shed light on standard baselines recently. By optimizing this loss term, the model imposes several assumptions/constraints on the decision boundary to avoid high-density regions of unannotated data.

#### Generative adversarial networks:

Moreover, generative adversarial learning can be adapted to semi-supervised learning for semantic segmentation [15–17] as well as by generating pseudo pixel-level predictions [18,19]. Adversarial networks use a critic to predict the pixel-level distribution of the data, which acts as an adversarial loss term with the goal to provide the generator with learnable useful visual features from the unlabeled data for medical image synthesis [20]. Nonetheless, learning high-dimensional data can be difficult. Autoencoders struggle with multi-modal data distributions, and generative models rely on computationally demanding models, which are especially difficult to train.

#### Mutual information estimation:

Recent work on representation learning has focused on mutual information estimation [21]. As mutual information maximization has been shown to be effective at capturing the salient attributes of data, being able to disentangle these attributes is another desirable property. For example, it may be beneficial to remove data attributes that are irrelevant to a given task, such as illumination conditions in object recognition.

#### Disentanglement learning:

Some newly introduced techniques have dedicated considerable attention to disentangle representation with generative modeling [22,23]. In disentangled representation, information is represented as a collection of (independent) factors [24], each of which corresponds to a meaningful aspect of the data [25,26]. A current line of research has argued that disentangled representations are beneficial for a variety of tasks, including (semi-)supervised learning of downstream tasks, few-shot learning [27], and exploratory medical data analysis. Additionally, these representations also make it easier for later processes to only use the relevant parts of the data as input.

#### Unpaired image-to-image translation:

Image-to-image translation was first proposed by Isola et al. in [28] in their conditional GAN paper. Furthermore, CycleGAN [29] tackles the problem of the above paired image translation approach by introducing a cycle-consistency loss to retrieve the original images by exploiting a cycle of translation. Later work [30] improved CycleGAN from one-to-one mapping to multimodal image generation. Nevertheless, in medical applications, image synthesis without explicit anatomy design constrain may lead to volatile anatomical structures and artifacts. Moreover, these methods are not aimed at medical image segmentation.

#### Domain Adaptation:

Domain adaptation, a form of transfer learning, encodes the distribution knowledge from a certain source domain to a different but related target domain, and thus, alleviates the domain shift discrepancy in real world applications [31]. Various methods have been proposed, including style and content-disentanglement [32], and adversary based approaches [33,34]. As described later, in this work, we disentangle the most interpretable segmentation-aware spatial (skeleton) information.

#### Normalization layers:

Inspired by instance normalization (IN) [35], conditional batch-normalization [36] and adaptive IN (AdaIN) [37] bring significant improvement in image generation. Later on, feature-wise linear modulation (FiLM) [38] and spatially adaptive denormalization (SPADE) [39] shed additional light over other normalization layers in image synthesis. In our proposed work, we also show how we can adapt both SPADE as well as FiLM normalization as part of a residual and common decoder, respectively.

#### Variational autoencoder-based models:

There have been several recent works involving disentangled learning with variational autoencoder (VAE) [24,40,41]. In contrast to these previous works, we will attempt to demonstrate the use of a VAE as a disentangled representation by sampling the sentiency code to separate the domain-specific information from the domain-invariant latent code.

### 1.3. Overview of the Proposed Method

To further address some of the shortcomings associated with existing methods, our efforts focus on learning meaningful spatial features utilizing a disentangler with a mutual information minimizer (MIM) to improve the adversarially trained generative models for improving semi-supervised segmentation and reconstruction results.

Our proposed method builds on several recent and key research findings in the fields of generative models, semi-supervised learning, and representation learning via disentanglement. We believe that the proposed framework’s reliance on as little as 1% labeled data for training, in concert with the high segmentation accuracy achieved, comparable to the fully or semi-supervised models, renders the proposed work an attractive solution for medical image segmentation, where access to vast expert-annotated data is expensive and often difficult to gain access to.

**Figure 1.**
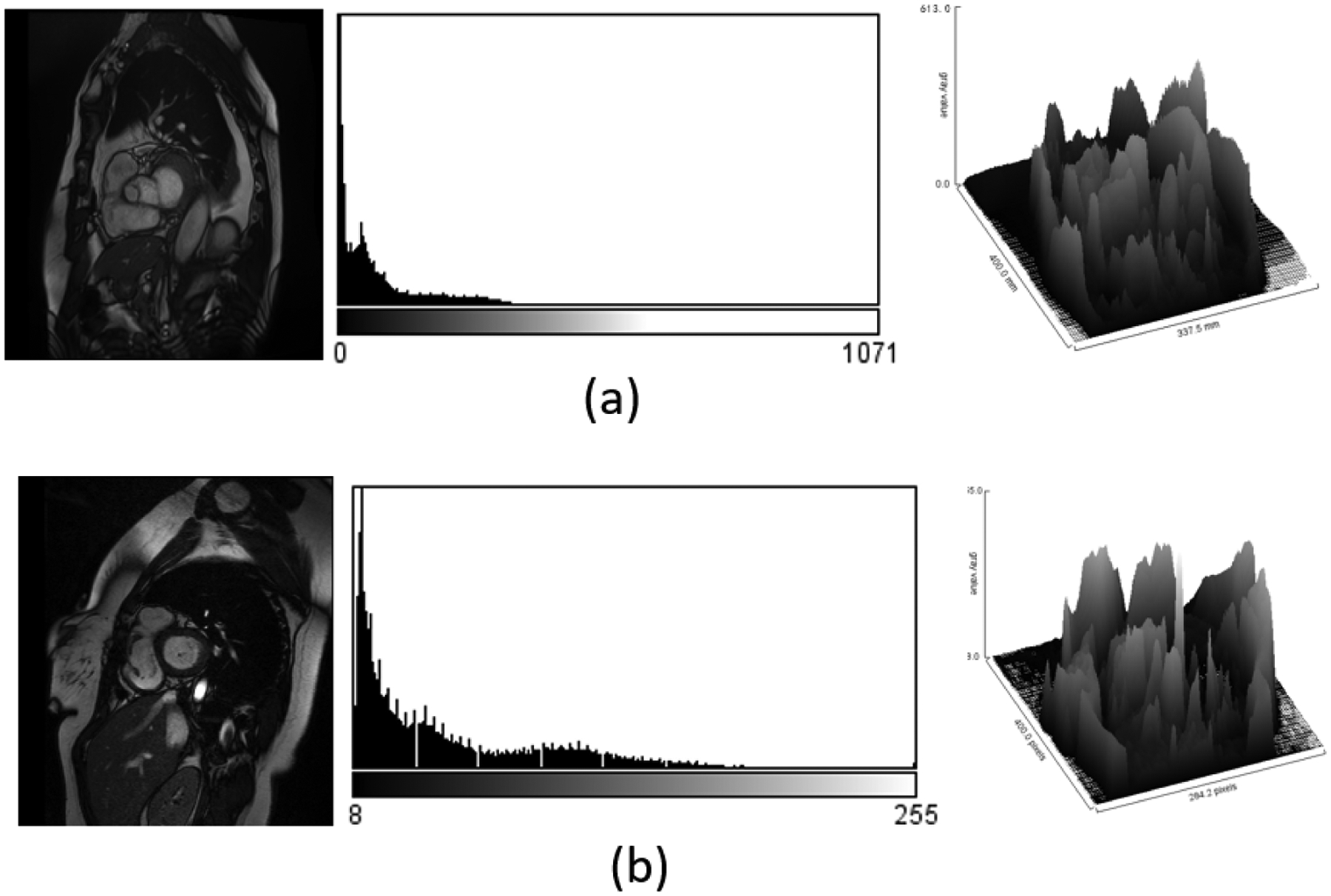
Images, histograms and surface plots of two 3D cardiac images featuring all slices of two random patients from the ACDC dataset are illustrated in (**a**,**b**). From left to right: cardiac MR image in 4 dimensions, histogram plot, and surface plot.

We approach this problem using a method that is based on disentangled representations and utilizes data from multiple scanners with varying intensities and contrast (Figure 1). Our method is intended to address multi-scanner unlabeled-data issues, such as intensity differences, and a lack of sufficient annotated data. Learning good data representations for medical imaging tasks ensures the preservation of relevant information and the removal of irrelevant information from the data to improve the interpretability of the learned features. Our model disentangles the input image into spatial and non-spatial space. These spatial features are represented as categorical feature maps, with each category corresponding to input pixels that are spatially similar and are from the same organ part. This semantic similarity aids in learning to be generalized the anatomical representation to any modality from different scanners. Furthermore, the non-spatial features capture the image’s global intensity information, which aids the renderer in painting the anatomy in the reconstructed image. Finally, because annotating data is time-consuming and expensive, the ability to learn this decomposition through disentanglement using a small number of labels is critical in medical image analysis.

In light of these needs, here we propose a semi-supervised (C*q*SL) model for learning disentangled representations that combines recent developments in semi-supervised learning–generative models and adversarial learning. We aim to factorize the representation of an image pair into two parts: a shared representation that captures the common information between images and an exclusive representation that contains the specific information of each image. Furthermore, in order to achieve representation disentanglement, we propose to minimize mutual information between shared and exclusive representations. Moreover, we use feature-wise linear modulation (FiLM) [38] to distinguish the domain-invariant information from the domain-specific information, as well as a spatially adaptive normalization (SPADE) [39]-based decoder to guide the synthesis of more texture information to restrain the posterior collapse of the VAE and spatial information.

**Figure 2.**
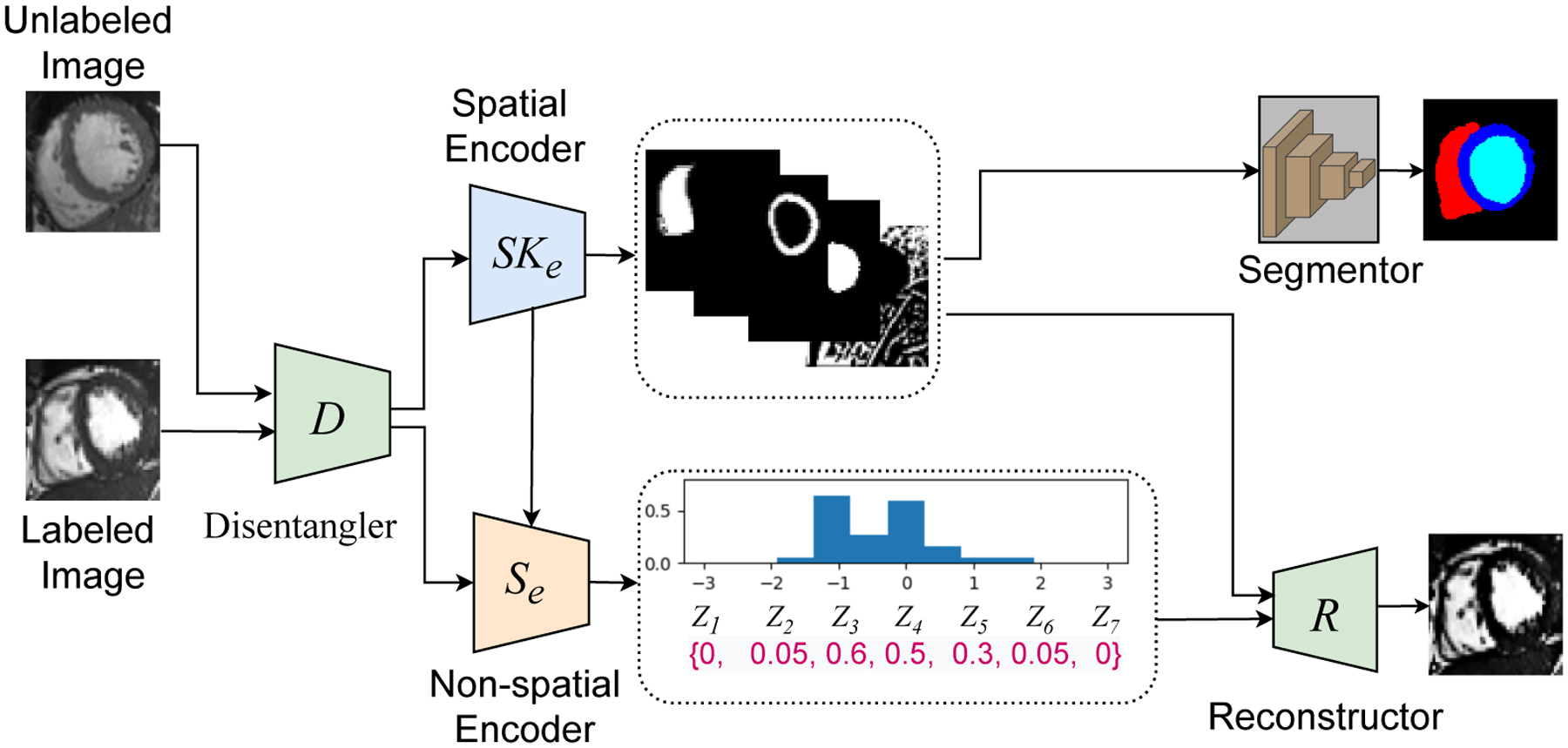
A simplified schematic overview of the proposed model.

To illustrate its adequacy, our model is applied to two of the foremost critical tasks in medical imaging—segmentation of cardiac structures and reconstruction of the original image—and both assignments are handled by the same model. Our model leverages a large amount of unannotated data from the ACDC (https://www.creatis.insa-lyon.fr/Challenge/acdc/databases.html, accessed on 2 October 2021) dataset to learn the interpretable representations through judicious choices of common factors that serve as strong prior knowledge for more complicated problems—the segmentation of cardiac structures. Figure 2 shows a simplified data view of our proposed model.

### 1.4. Contributions

Our proposed work makes several contributions summarized as follows:

1. We combine recent developments in disentangled representation learning with strong prior knowledge about medical imaging data that features a decomposition into “skeleton (spatial)” and “sentiency (non-spatial)”, to ensure that the spatial information is not mixed up with the non-spatial information.
2. We alter the usual cross-entropy loss to down-weigh the loss applied to well-classified samples in order to overcome the foreground–background class imbalance problem. Specifically, we exploit a novel supervised loss—the weighted-soft-background-focal (WSBF) loss, which focuses the training on a set of hard examples to ensure that this loss can differentiate between easy/hard examples.
3. We employ both qualitative and quantitative tests to evaluate the usefulness of our framework, which show that our model outperformed fully supervised methods, even when using only 1% labeled data for training.

The paper is organized as follows: Section 1 establishes the general background and motivation of the work, reviews the related literature on latest developments in the field of domain adaptation, semi-supervised learning and representation learning, and provides an overview of the proposed work; Section 2 describes our proposed methodology; Section 3 presents our quantitative and qualitative results achieved using our proposed method for both image segmentation and reconstruction, along with the associated ablation studies; Section 4 concludes the paper with a summary of our contributions and promising future research directions.

## 2. Methods

### 2.1. CqSL Model Overview

We propose a model that combines the concept of variational generative and adversarial learning, and disentangled interpretation learning in a semi-supervised learning scheme, which is suited for domain-adapted segmentation as well as reconstruction.

We define the learning task as follows: given an (unknown) data distribution p(x,y) over images and segmentation masks, we define a source domain having a training set, ${𝒟}_{ℒ}={\left\{\left(x_{i}^{l},y_{i}^{l}\right)\right\}}_{i=1}^{n_{l}}$ with $n_{l}$ labeled examples, and another domain having a training set, ${𝒟}_{𝒰ℒ}={\left\{\left(x_{j}^{ul}\right)\right\}}_{j=1}^{n_{ul}}$ with $n_{ul}$ unlabeled examples, which are sampled as independent, identically distributed variables from p(x,y) and p(x) distribution. Empirically, we want to minimize the target risk $\in_{t}(\phi ,\theta )={min}_{\phi ,\theta }\;\;{ℒ}_{ℒ}\left({𝒟}_{ℒ},(\phi ,\theta )\right)+\gamma {ℒ}_{𝒰ℒ}\left({𝒟}_{𝒰ℒ},(\phi ,\theta )\right)$, where ${ℒ}_{ℒ}$ is the supervised loss for segmentation, ${ℒ}_{𝒰ℒ}$ is the unsupervised loss defined on unlabeled images and ϕ,θ denotes the learnable parameters of the overall network.

**Figure 3.**
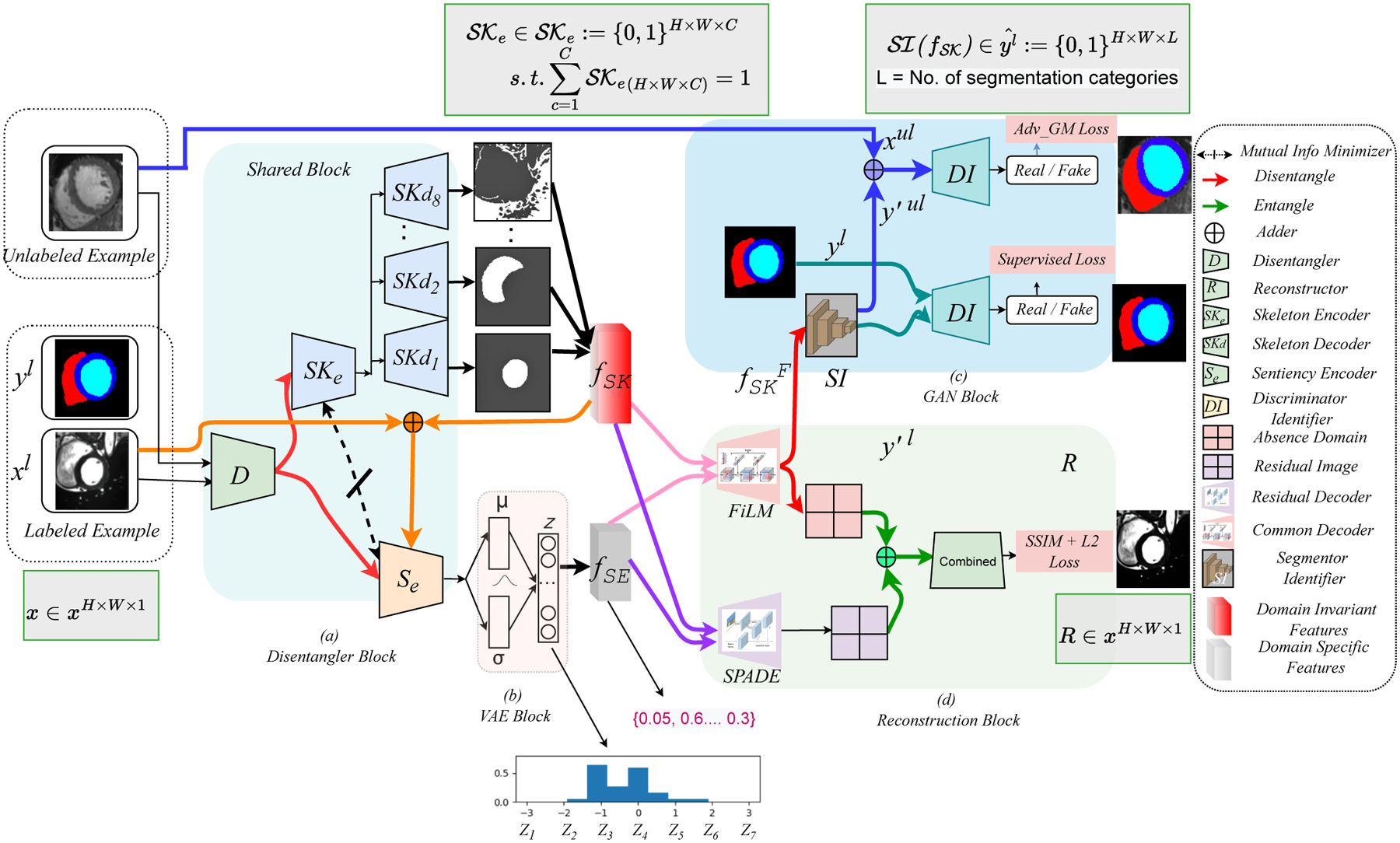
Illustration of ***CqSL* framework**: Our model makes use of both labeled as well as unlabeled images. The first block (**a**) crops the input images to a specific dimension. Then, we disentangle the latent features of the images via a disentangled block. An input image is first encoded to a multi-channel spatial representation, $SKd_{n=1,2\ldots 8}$. Then, $SKd_{n}$ can be fed into a segmentation network *SI* to generate a multi-class segmentation mask. (**c**) We train a generative network, which predicts semantic labels for both labeled and unlabeled data. (**b**) A sentiency encoder $S_{e}$ uses the factor $SKd_{n}$ and the input image to generate a latent vector z representing the imaging modality using a variational autoencoding block. (**d**) The decoder networks combine the two representations $SKd_{n}$ and z to reconstruct the input image.

We propose to solve the task by learning domain-specific and domain-invariant features that are discriminative of the semgentor and reconstructor. Figure 3 shows the proposed model comprised of five components—(1) disentanglement component, (2) a disentangled variational autoencoder (DVAE), (3) a mask segmentor identifier (SI), (4) a mask discriminator identifier (DI), and (5) a reconstructor R.

**Figure 4.**
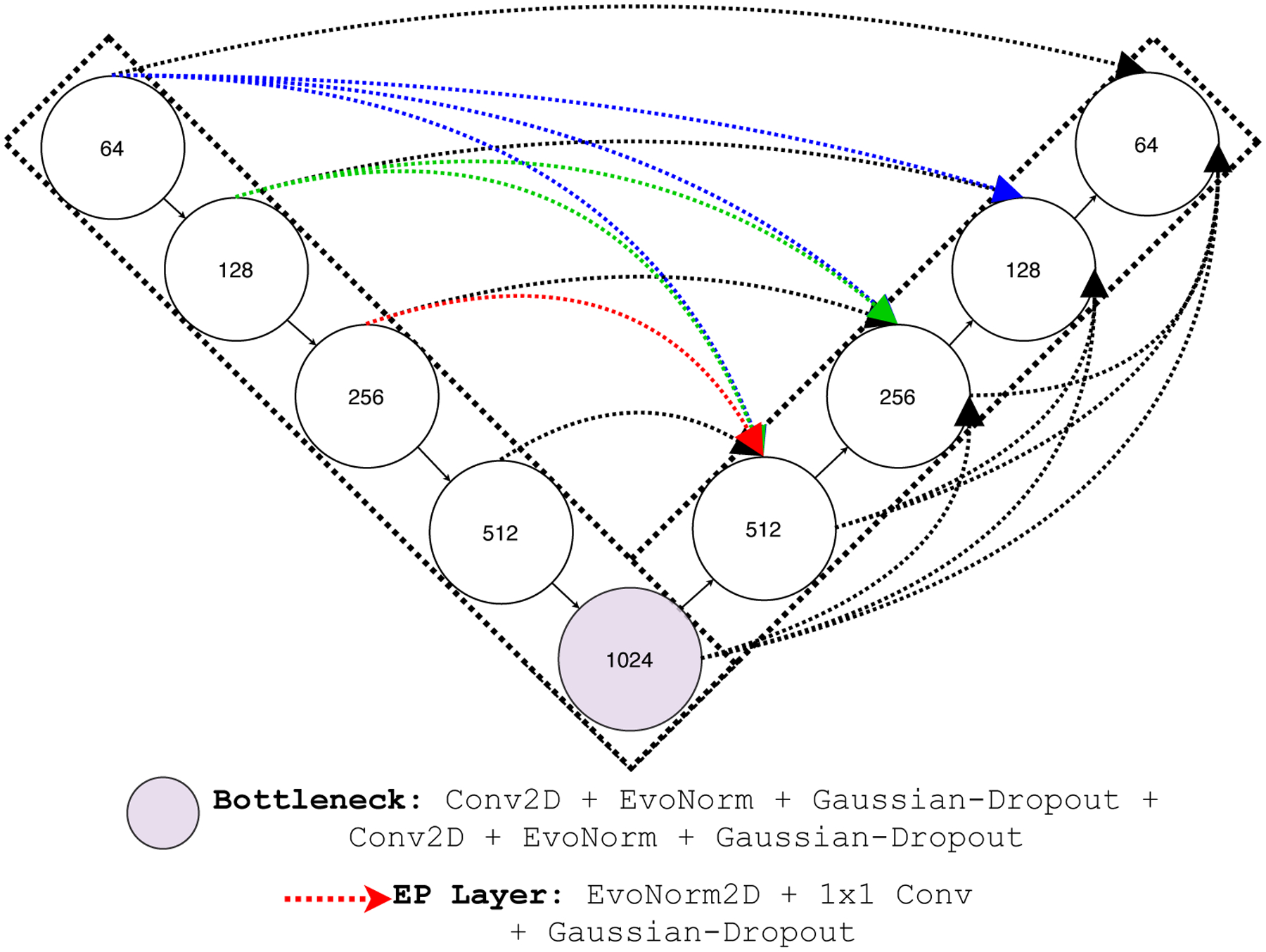
Illustration of EPU-Net++ Block: skip connections are replaced with a long projection block.

The disentangler D (Figure 3a) is designed to factorize the representation of an image pair into two parts: a shared spatial representation (skeleton, ${SK}_{e}$) that captures the common information between images and an exclusive non-spatial representation (sentiency, $S_{e}$) that contains the specific information of each image. The skeleton block $SK_{e}$ is a modified U-Net++ [42] type architecture (EPU-Net++) (Figure 4 and Section 2.1.1) and is responsible for capturing the domain-invariant features $\left(f_{SK}\right)$. The sentiency block $S_{e}$ is a DVAE (Figure 3b) type architecture, which takes both the input image and the domain-invariant features $\left(f_{SK}\right)$ as the input to map domain-specific features $\left(f_{SE}\right)$ using the reparameterized trick [43].

The reconstruction block consists of two decoders: the SPADE-based decoder takes the $\left(f_{SE}\right)$ feature from the sentiency block and proceeds directly to the reconstructor R (Figure 3d), while the FiLM-based decoder works as another disentangler, which untangles a segmentor identifier (*SI*) (Figure 3c), used for segmentation and extracted features, which then proceed directly to the reconstructor R. The reconstructor R aims to recover the original image from both ($\left.f_{SK},f_{SE}\right)$. A mutual information minimizer (Figure 3a block) is applied between $\left(SK_{e\;}\;\text{and}\;\left.S_{e}\right)\right.$ to enhance the disentanglement. A supervised trainer is trained on the labeled data to predict the segmentation mask distribution optimizing a supervised loss. An unsupervised trainer is trained on the unlabeled data, optimizing unsupervised losses (Algorithm 1 specifies the overall training procedure). Both the unsupervised and supervised trainers share the same block, as mentioned above.


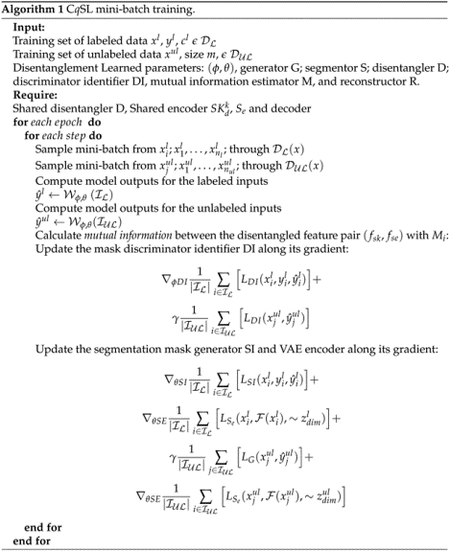


#### 2.1.1. Disentanglement

**Figure 5.**
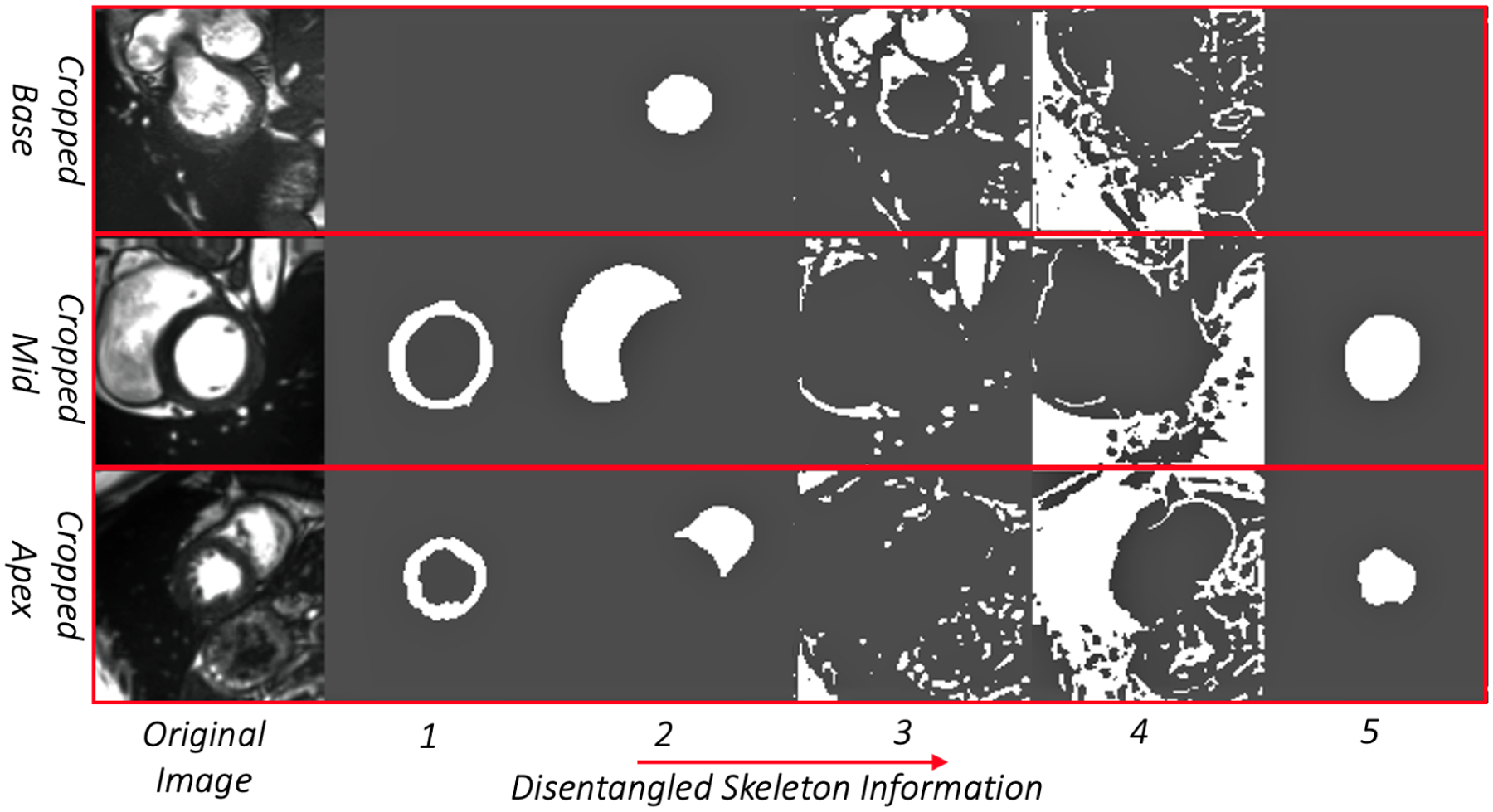
Representative examples showing the 5 (out of 8) most semantic disentangled multi-channel binary maps of the spatial information generated from the skeleton decoder from the base to apex (top to bottom rows). Some channels indicate anatomical portions that are well-defined, such as the myocardium, left ventricle or the right ventricle, while others represent the remaining anatomy needed to characterize the input image.

Referring to Figure 3a, the disentangler block factorizes the image features into spatial (skeleton/physique) features, as well as non-spatial (sentiency) features that carry residual information. The skeleton block is a modified U-Net type architecture—EvoNorm-Projection-UNet++ (EPU-Net++) as shown in Figure 4. We attach eight different decoders at the common bottleneck layer of EPU-Net++. Each decoder captures bottleneck features from 2D cropped images and transforms them into different feature maps consisting of a number of binary channels which are then combined together to form eight most effective channels: $x_{ST}\overset{(0,1{)}_{\left(h\times w\times c\right)}}{⟶}\left\{\sum_{i=1}^{i=8}\;f_{SK_{i}}\right\}$. These feature maps are responsible for capturing the domain-invariant features and contain cardiac structures (myocardium, the left and the right ventricle), effective for segmentation and some surrounding structures, effective for reconstruction (Figure 5).

We use a separate neural network for capturing the sentiency information i.e., domain-specific information. We combine the crop image and the domain-invariant features to penalize the deviation of latent features from the prior distribution employing *Kullback*–*Leibler* divergence by applying a VAE architecture (Figure 3b) with the following objective function:

(1) $$L_{vae}=\sum \;\left|\left(p\left(z_{i}\right)\text{log}\;\frac{p\left(z_{i}\right)}{p\left(z_{i}|x_{i}^{ul},f_{SK_{i}}\right)}\right)\right|$$

A VAE learns a low dimensional latent space such that the acquired latent representations fit a prior distribution that is predetermined to be an isotropic multivariate Gaussian p(z)=𝒩(0,1). An encoder and a decoder make up a VAE. Given an input, the encoder guesses the Gaussian distribution’s parameters. In order to enable learning through back propagation, this distribution is then sampled using the reparameterization technique, and the resulting sample is sent through the decoder to reconstruct the input.

We use disentangled features as the prior distribution in a VAE (Equation (1)) to remove class-irrelevant features (e.g., background pixels) and ensure that domain-invariant features are well-disentangled from class-specific features, because the image-only a priori aligns the latent features to a normal distribution.

#### 2.1.2. Mutual Information Minimizer

To better exploit the disentanglement, we add a regularization term based on mutual information (MI), denoted as MIM, which measures the “amount of information” learned from knowledge of random variable Y about the other random variable X [44]. In this paper, we adopt the *mutual information neural estimator (MINE*) [45], $MI\left(f_{SK},f_{SE}\right)$:

(2) $$\frac{1}{N}\sum_{i=1}^{N}\;M(\alpha ,\beta ,\theta )-\text{log}\;\left(\frac{1}{N}\sum_{i=1}^{N}\;\;{\text{exp}}^{M\left(\alpha ,\beta^{\prime },\theta \right)}\right)$$

where (α,β) are sampled from the joint distribution of $\left(f_{SK},f_{SE}\right)$ and $\beta^{\prime }$ is sampled from the marginal distribution.

The mutual information can be expressed as the difference of two entropy terms MIM (X;Y)=H(X)-H(X∣Y); we seek to minimize the MI between domain-invariant and domain-specific features $\left(f_{SK},f_{SE}\right)$, whereas we make an assumption that the information content does not vary much between intra-domains (Figure 3a).

#### 2.1.3. Segmentation

The mask segmentor identifier (*SI*) (Figure 3c) takes the output from the FiLM decoder $f_{SK}^{F}$ as input and generates predicted segmentation mask $SI\left(f_{SK}\right)={\overset{ˆ}{y}}^{l}\in \{0,1{\}}^{\left(H\times W\times L\right)}$, where L is the number of categories (RV, LV, LV-Myo, and background) in the training dataset. We exploit a novel supervised loss, weighted soft background focal (WSBF) loss, $L_{SI(\text{seg})}^{ℒ}={ℒ}_{\text{WSFL}}+{ℒ}_{BFD}$ for the base model, which is a combination of background focal dice loss (BFD) and weighted soft focal loss (WSFL):

(3) $$L_{SI(\text{seg})}^{ℒ}=\left[\alpha_{0}+y\left(\alpha_{1}-\alpha_{0}\right)\right]|y-\overset{ˆ}{y}{|}^{\gamma }\cdot w_{\text{map }}\cdot CE(y,\overset{ˆ}{y})+\;\sum_{c}\;\;{\left[2-\frac{2\sum \;y\overset{ˆ}{y}+\epsilon }{\sum \;(y+\overset{ˆ}{y})+\epsilon }-\frac{2\sum \;\overset{‾}{y}\overset{-}{\overset{ˆ}{y}}+\epsilon }{\sum \;(\overset{‾}{y}+\overset{-}{\overset{ˆ}{y}})+\epsilon }\right]}^{\frac{1}{\gamma }}$$

where $\alpha_{0}$ and $\alpha_{1}$ are designed to account for class imbalance and are treated as hyperparameters, the term $|y-\overset{ˆ}{y}{|}^{\gamma }$ is used to down-weigh examples with backgrounds, where γ varies in the range [1,3]. The term $CE(y,\overset{ˆ}{y})=-y\text{log}\;\overset{ˆ}{y}-(1-y)\text{log}\;(1-\overset{ˆ}{y})$ denotes the cross-entropy loss.

On the other hand, the data with no corresponding segmentation masks are trained by minimizing the unsupervised loss via a *KL* divergence based on least-squares GAN [46]. However, since the least-squares loss is not sufficiently robust, we introduce a new divergence loss function by incorporating it into a Geman–McClure model [47] fashion called *adversarial-Geman*–*McClure (adv-GM*) loss between the ground truth of real mask $y^{l}$ and prediction on unlabeled data $y^{ul}$:

(4) $$L_{SI(adv-GM)}^{𝒰}=\frac{DI{\left(SI\left(f_{SK}\left(x^{ul}\right)\right)\right)}^{2}+{\left(DI\left({\overset{ˆ}{y}}^{ul}\right)-1\right)}^{2}}{2\beta +DI{\left(SI\left(f_{SK}\left(x^{ul}\right)\right)\right)}^{2}+{\left(DI\left({\overset{ˆ}{y}}^{ul}\right)-1\right)}^{2}}$$

where β is the scale factor which varies in the range of [0, 1] and we set β=0.5 in our experiment.

#### 2.1.4. Image Reconstruction

**Figure 6.**
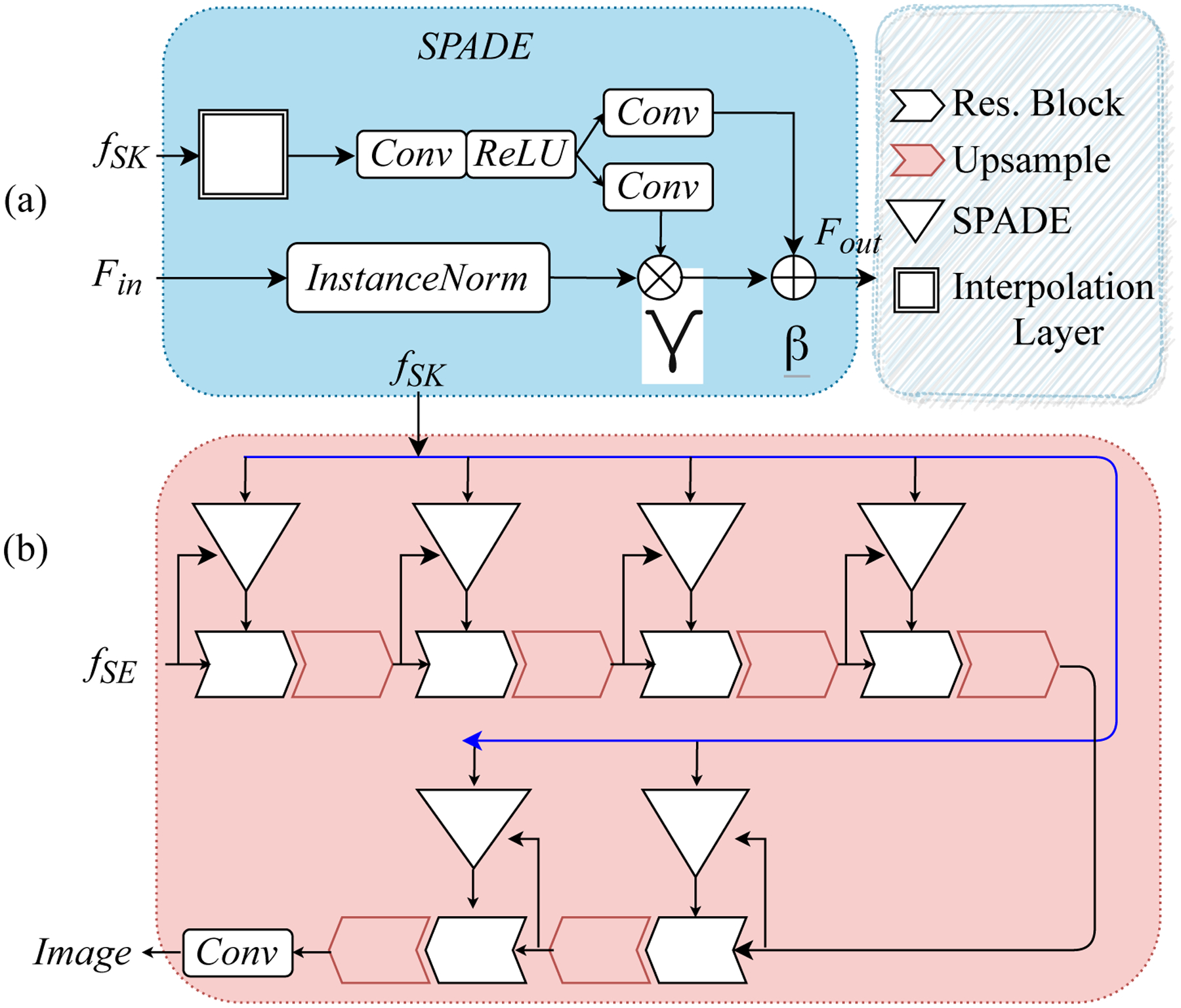
Detailed architecture of SPADE block: (**a**) shape-aware normalization block where the spatial tensors, γ and β are multiplied and added to the input features; (**b**) decoder block $f_{SES}$ with shape-aware normalization.

To better capture the anatomical shape and the intensity information in the synthetic image, we propose a two-branched reconstruction architecture featuring two separate decoders: one is conditioned with FiLM [38], and the other with SPADE [39] (Figure 6a) and both are then concatenated to produce a realistic image. The FiLM decoder consists of multiple FiLM layers, a gamma-beta predictor, and convolutional layers with 3×3 kernel and (8, 8, 8, 8, 1) channels in the stride of 1. Each convolution layer is followed by batch normalization layer along with a Leaky-ReLU layer.

To better retain the non-spatial information in the MR image, we integrate the shape knowledge into the idea of SPADE [39] and form a shape-aware normalization layer (see Figure 6). SPADE first normalizes the input feature $F_{in}$ with a scale α and a shift μ learned from sampled z using an instance-normalization (InstanceNorm) layer, inspired by [38] and then denormalizes it based on a spatial representation $f_{SK}$ through learnable parameters γ and $\beta .f_{SK}$ is then interpolated to match the texture dimension of the sampled z from the sentiency encoder and used as a semantic mask for SPADE:

(5) $$F_{\text{out }}=\frac{F_{in}-\mu }{\alpha }\times \gamma \left(f_{SK}\right)+\beta \left(f_{SK}\right)$$

where $F_{in}$ and $F_{\text{out}}$ denote the output feature maps. γ and β are learned from $f_{SK}$ by three Conv layers. Thus, the learned shape information precludes washing away the anatomical information, which encourages the image synthesis to be more accurate. The first convolution layer inside the SPADE block (Figure 6) encodes the interpolated $f_{SK}$, and the other two convolution layers learn the spatial tensors γ and β. Simultaneously, an instance normalization layer is applied to the intermediate feature map, which is then modulated by the scale and shift parameters γ and β learned from sampled z to produce the output. Finally, the output of the two decoders is re-entangled in order to reconstruct an image.

### 2.2. Objective Functions

The training objective function consists of multiple losses for labeled and unlabeled data, each weighted by some scalar term λ:

(6) $$L_{\text{total }}=\lambda_{\text{seg }}L_{SI\left(seg\right)}^{ℒ}+\lambda_{\text{adv-GM }}\{L_{SI,DI\left(adv-GM\right)}^{ℒ}\;+L_{SI,DI^{u}\left(adv-GM\right)}^{𝒰}\}+\lambda_{\text{vae }}L_{vae}\;\;+\lambda_{SL_{2}SIM}\left\{L_{SL_{2}SIM}^{ℒ}+L_{SL_{2}SIM}^{𝒰}\right\}\;+\lambda_{MIM}\text{MIM}\left(f_{SK},f_{SE}\right)$$

where $\lambda_{t}$ is the weight for the loss of type t. In this paper, we empirically set the weights as $\lambda_{\text{vae}}=0.01,\lambda_{\text{seg}}=10,\lambda_{\text{adv–GM}}=10,\lambda_{SL_{2}SIM}=0.01,\lambda_{\text{MIM}}=1$.

#### 2.2.1. Segmentation Loss

Since the model is trained on both labeled and unlabeled data, the segmentation loss $L_{\text{seg}}$ includes both supervised and unsupervised losses:

(7) $$L_{seg}=L_{sup}+L_{\text{usup}}$$

##### Supervised Loss.

Our supervised cost is based on the combination of the two following functions: (1) the weighted soft focal loss, and (2) the background focal dice loss mentioned in Equation (3) $\left(L_{sup}=L_{SI(seg)}^{ℒ}\right)$.

##### Unsupervised Loss.

The discriminator identifier is adversarially trained for the labeled and unlabeled data and updated along with adversarial-Geman–McClure (adv-GM) loss $L_{\text{usup}}=L_{SI,DI(adv-GM)}^{ℒ}+L_{SI,DI^{u}(adv-GM)}^{𝒰}$. For labeled data, the adversarial loss is

(8) $$L_{SI,DI\left(adv-GM\right)}^{ℒ}=\;\frac{E_{x\sim x_{i}^{l}}\left[DI{\left(SI\left(f_{SK_{i}}\left(x_{i}^{l}\right)\right)\right)}^{2}\right]+E_{y\sim y_{i}^{l}}\left[{\left(DI\left(y_{i}^{l}\right)-1\right)}^{2}\right]}{2\beta +E_{x\sim x_{i}^{l}}\left[DI{\left(SI\left(f_{SK_{i}}\left(x_{i}^{l}\right)\right)\right)}^{2}\right]+E_{y\sim y_{i}^{l}}\left[{\left(DI\left(y_{i}^{l}\right)-1\right)}^{2}\right]}$$

Similarly, for the unlabeled data, the adversarial loss is

(9) $$L_{SI,DI^{u}\left(adv-GM\right)}^{𝒰}=\frac{E_{x\sim x_{i}^{ul}}\left[DI^{u}{\left(SI\left(f_{SK_{i}}\left(x_{i}^{ul}\right)\right)\right)}^{2}\right]}{2\beta +E_{x\sim x_{i}^{ul}}\left[DI^{u}{\left(SI\left(f_{SK_{i}}\left(x_{i}^{ul}\right)\right)\right)}^{2}\right]}\;\frac{+E_{y\sim {\hat{y}}_{i}^{ul}}\left[{\left(DI^{u}\left(y_{i}^{ul}\right)-1\right)}^{2}\right]}{+E_{y\sim {\hat{y}}_{i}^{ul}}\left[{\left(DI^{u}\left(y_{i}^{ul}\right)-1\right)}^{2}\right]}$$

##### VAE Loss.

For the smooth texture detail of the input data, the VAE learns factorized representations to optimize a KL-divergence loss, given an image $x_{i}^{ul}$, and its decomposed skeleton feature $f_{SK}$ (Equation (1)).

#### 2.2.2. Reconstruction Loss

We adopt a novel reconstruction loss as a combination of structural similarity (SSIM) and *L*_2_ loss–*SL*_2_*SIM* in order to enforce the similarity between recovered image and original image for better learning the distribution of images.

##### *SL*_2_SIM Loss.

Since the image intensities vary across imaging scanners, as a result, there are high chances that the generative model will tend to *mode collapse*. This structural $L_{2}$ similarity ($\left.{\text{S}L}_{2}\text{SIM}\right)$ loss provides a similarity measure between the input image and the reconstructed image based on high light-dark variance, contrast, and structural similarity. The concatenated FiLM and SPADE decoder learn the parameters to reconstruct the input image using a novel combination of structured similarity loss and $L_{2}$ loss. For labeled data, the reconstruction loss is

(10) $$L_{SL_{2}SIM}^{ℒ}=E_{x_{i}\sim x_{i}^{l}}[1-SL_{2}SIM\{x_{i}^{l},(ℱ\left(f_{SK_{i}},f_{SE_{i}}\right)\;⊕𝒮\left(f_{SK_{i}},f_{SE_{i}}\right))\}+\alpha \overset{n_{l}}{\underset{i=1}{\sum }}∥\{x_{i}^{l}-(ℱ\left(f_{SK_{i},},f_{SE_{i}}\right)\;⊕𝒮\left(f_{SK_{i},}f_{SE_{i}}\right))\}{∥}_{2}^{2}]$$

Similarly, for unlabeled data, the reconstruction loss is

(11) $$L_{SL_{2}SIM}^{𝒰}=E_{x_{i}\sim x_{i}^{ul}}[1-{\text{SL }}_{2}SIM\{x_{i}^{ul},(ℱ\left(f_{SK_{i}},f_{SE_{i}}\right)\;⊕𝒮\left(f_{SK_{i}}f_{SE_{i}}\right))\}+\alpha \overset{n_{ul}}{\underset{i=1}{\sum }}∥\{x_{i}^{ul}-(ℱ\left(f_{SK_{i}},f_{SE_{i}}\right)\;⊕𝒮\left(f_{SK_{i}},f_{SE_{i}}\right))\}{∥}_{2}^{2}]$$

where $\text{S}L_{2}\text{SIM}$ is the structure similarity index term and α is a regularized term.

### 2.3. Experiments

#### 2.3.1. Datasets

We validate the effectiveness of C*q*SL on a widely adopted cardiac image segmentation challenge dataset by conducting several comparisons to other baseline models. We use the STACOM 2017 *Automated Cardiac Diagnosis Challenge* (ACDC) dataset (https://www.creatis.insa-lyon.fr/Challenge/acdc/databases.html, accessed on 2 October 2021), consisting of short-axis cardiac cine-MR images acquired for 100 patients (1920 labeled and 23,530 unlabeled images) divided into 5 subgroups: normal (NOR), myocardial infarction (MINF), dilated cardiomyopathy (DCM), hypertrophic cardiomyopathy (HCM), and abnormal right ventricle (ARV), available through the 2017 MICCAI-ACDC STACOM challenge [48]. The images were acquired over a 6 year period using two MRI scanners of different magnetic strengths (1.5 T and 3.0 T). The images were acquired using the SSFP sequence with spatial resolution 1.37 to 1.68 mm^2^/pixel and 28 to 40 frames per cardiac cycle. We split the dataset into three sets—training (70), validation (15), and test (15).

#### 2.3.2. Implementation Details

##### Input:

**Figure 7.**
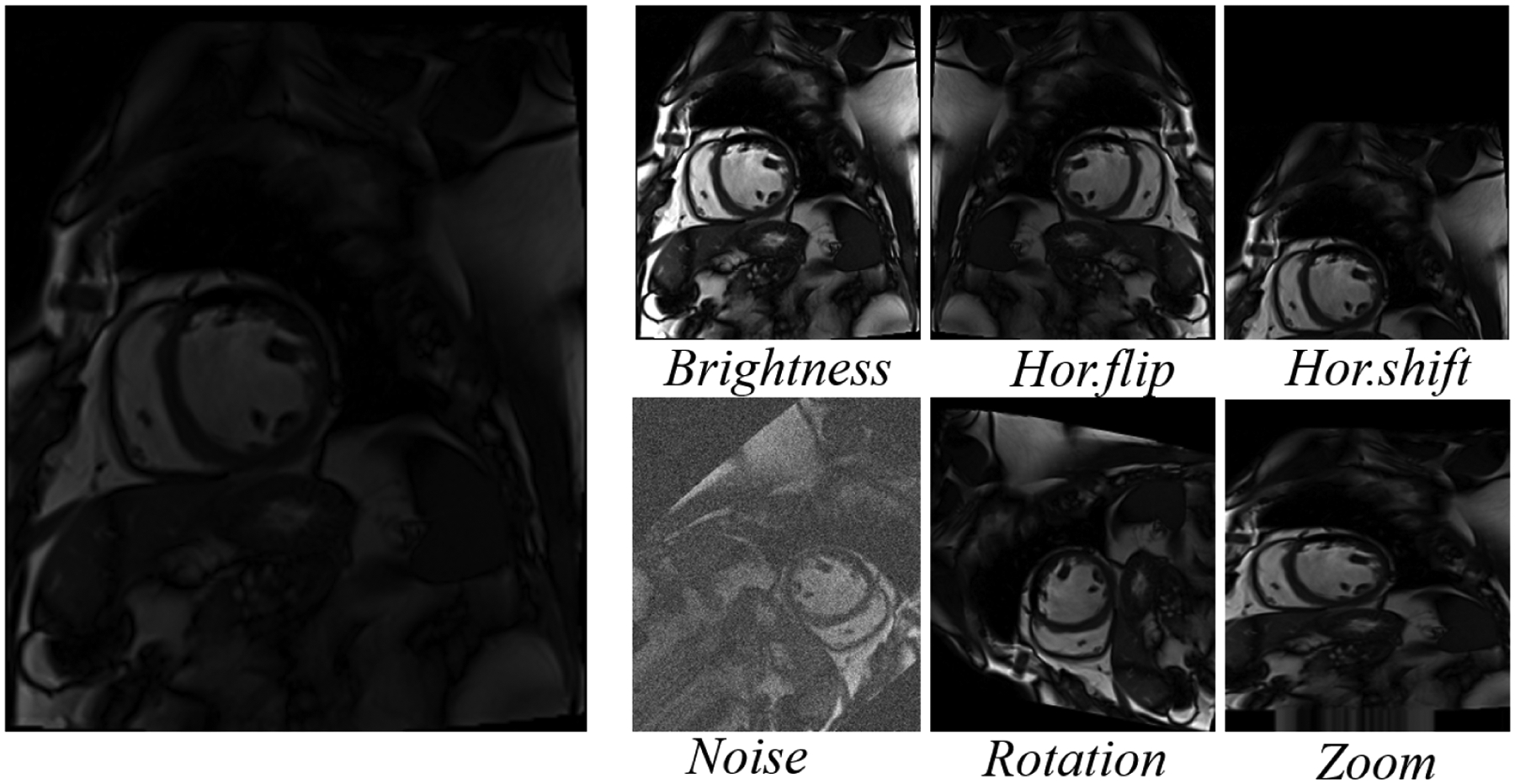
Example images of applying data augmentation via affine transformations.

All the cine cardiac images employed slice-wise normalization in the range [0, 1] by subtracting the mean slice intensity from each pixel intensity, then dividing it by the difference between the maximum and minimum slice intensity. All images were resampled to 1.37 mm^2^/pixel. Images were cropped to 192×192×1 pixels before feeding to the models. We applied data augmentation on-the-fly during training as shown in Figure 7, which includes random rotations up to 90 degrees, random zooms up to 20%, random horizontal shifts up to 20%, random horizontal and/or vertical flips, and noise addition (Figure 7).

##### Baselines Architecture:

As the disentangled encoder in the skeletal block, we use a modified U-Net-like architecture, EPU-Net++, and as a sentiency encoder, we use VAE. As the reconstruction block, we use FiLM- and SPADE-based decoder as used in [49].

##### Generator-Discriminator Network:

Our segmentation generator network consists of 3 convolution layers with 3×3 kernel and {64, 64, 1} channels in the stride of 1. Each convolution layer is followed by a batch normalization [50] layer along with a Leaky-ReLU [51] except the last layer. We use the structure similar to DCGAN [52] for the discriminator network.

##### EvoNorm-Projection skip connections:

In our skeleton encoder, we replace the standard skip connection with a normalized-projection operation using EvoNorm2D+1×1-Conv+Gaussian–dropout, as in Figure 4. This new normalization layer adds together two types of statistical moments–batch variance, and instance variance, both of which capture both the global and local information across images without having any explicit activation function [53]. The proposed projection operation helps in reducing the learnable weights and also allows intricate learnability of cross-channel information.

##### Additional Factors:

The performance of semi-supervised models trained for image segmentation can be significantly impacted by the proper selection of regularizer, optimizer, and hyper-parameters. The model implemented in Keras was initialized with the He normal initializer and trained for 100 epochs with a batch size of 4. We trained all the components iteratively with the Adam optimizer with a 0.0001 learning rate to minimize the objective function. All experiments were conducted on a machine equipped with two NVIDIA RTX 2080 Ti GPU (each 11GBs memory). The detailed training procedure is presented in Algorithm 1.

##### Training:

In our semi-supervised setup, we trained the network on varying proportions of labeled data: 1%, 10%, 20%, 30%, 50%, and 90% as a labeled set and used the rest of the data as the training unlabeled set to hold $\left|{𝒟}_{ℒ}\right|\leq \left|{𝒟}_{𝒰ℒ}\right|$. In Section 3, we include an ablation study to investigate the importance of adding different loss components in our model C*q*SL which is comprised of all the three loss functions: WSBF, MIM, Adv-GM. (Definitions are provided in Sections 2.1.2 and 2.1.3.)

We experimented an ablation study containing four of the variants of our proposed model C*q*SL. The variants are described as follows: ${\;}^{1}\text{C}q\text{SL}$, without weighted-soft focal loss (WSFL); $\;{\;}^{2}\text{C}q\text{SL}$, without adversarial-Geman–McClure loss (Adv-GM); $\;{\;}^{3}\text{C}q\text{SL}$, dice and cross-entropy loss only; and ${\;}^{4}\text{C}q\text{SL}$, without mutual information minimizer loss (MIM). Here, we utilize the same backbones as the baselines with the only exceptions being different loss functions. To clarify our point, in ${\;}^{1}\text{C}q\text{SL}$, we removed the weighted soft focal loss (WSFL) from the weighted soft background focal loss (WSBF), while keeping the background focal dice loss (BFD), mutual information minimizer loss (MIM) and adversarial-Geman–McClure (adv-GM) the same as before. In ${\;}^{2}\text{C}q\text{SL}$, we removed our Geman–McClure version of adversarial loss, while keeping the regular adversarial loss, weighted soft background focal loss (WSBF), and mutual information minimizer loss (MIM) the same as before. Similarly, in ${\;}^{3}\text{C}q\text{SL}$, we used *DICE* + *CE* loss rather than using our novel weighted soft background focal loss (WSBF) while keeping the mutual information minimizer loss (MIM) and adversarial-Geman–McClure (adv-GM) the same as before. Finally, in ${\;}^{4}\text{C}q\text{SL}$, we removed our mutual information minimizer loss (MIM) loss, while keeping the weighted soft background focal loss (WSBF), and adversarial Geman–McClure (adv-GM) the same as before. Additionally, the sentiency block, $S_{e}$ and the skeleton block, $SK_{e}$ were in place. We evaluated the performance of all four C*q*SL semi-supervised variants as summarized in Tables 1–3 in the Results section, and, as illustrated later, the ${\;}^{1}\text{C}q\text{SL}$ variant performed best, but for the sake of consistency, we asses and compare the performance of all four implemented variants.

### 2.4. Evaluation Metrics

To evaluate the performance of the semantic segmentation of cardiac structures, we use the standard metrics, including Dice score, Jaccard index, Hausdorff distance (HD), precision (Prec), and recall (Rec).

#### 1. Dice and Jaccard Coefficients:

The Dice score is used to measure the percentage of overlap between manually segmented boundaries and automatically segmented boundaries of the structures of interest. Given the set of all pixels in the image, set of foreground pixels by automated segmentation $S_{1}^{a}$, and the set of pixels for ground truth $S_{1}^{g}$, the Dice score can be compared with $\left[S_{1}^{a},S_{1}^{g}\right]\subseteq \Omega$, when a vector of ground truth labels $T_{1}$ and a vector of predicted labels $P_{1}$ as

(12) $$\text{Dice}\;\left(T_{1},P_{1}\right)=\frac{2\left|T_{1}\cap P_{1}\right|}{\left|T_{1}\right|+\left|P_{1}\right|}$$

The Dice score will measure the similarity between two sets, $T_{1}$ and $P_{1}$, and $\left|T_{1}\right|$ denotes the cardinality of the set $T_{1}$ with the range of $D\left(T_{1},P_{1}\right)\epsilon [0,1]$.

The Jaccard index or Jaccard similarity coefficient is another metric which aids in the evaluation of the overlap in two sets of data. This index is similar to the Dice coefficient but mathematically different and typically used for different applications. For the same set of pixels in the image, Jaccard index can be written by the following expression:

(13) $$\text{Jaccard}\;\left(T_{1},P_{1}\right)=\frac{\left|T_{1}\cap P_{1}\right|}{\left|T_{1}+P_{1}\right|}$$

#### 2. Precision and Recall

Precision and recall are two other metrics used to measure the segmentation quality which are sensitive to under- and over-segmentation. High values of both precision and recall indicate that the boundaries in both segmentation agree in location and level of detail. Precision and recall can be written as

(14) $$\text{Precision}=\frac{TP}{TP+FP}$$

(15) $$\text{Recall}=\frac{TP}{TP+FN}$$

where *TP* denotes true positive rate when a prediction-target mask pair has a score which exceeds some predefined threshold value; *FP* denotes the false positive rate when a predicted mask has no associated ground truth mask; and *FN* denotes the false negative rate when a ground truth mask has no associated predicted mask.

#### 3. Hausdorff distance (HD):

Hausdorff distance (HD) measures the maximum distance between the two surfaces. Let $S_{A}$ and $S_{B}$ be surfaces corresponding to two binary segmentation masks, A and B, respectively. The Hausdorff distance (HD) is defined as

(16) $$HD=\text{max}\left(\underset{p\in S_{A}}{\text{max}}\;d\left(p,S_{B}\right),\underset{q\in S_{B}}{\text{max}}\;d\left(q,S_{A}\right)\right)$$

where d(p,S)=minqϵSd(p,q) is the minimum Euclidean distance of point p from the points q ϵ S.

#### 4. Image Quality Metrics:

##### PSNR:

The peak signal-to-noise ratio (PSNR) is the most commonly used quality assessment technique for determining the quality of lossy image compression codec reconstruction. The signal is the original data, and the noise is the error caused by the distortion.

#### 5. Clinical Indices:

To assess the performance of the ventricles, different indices have been used in the literature [54], such as left ventricular volume (LVV), left ventricular myocardial mass (LVM), stroke volume (SV), and ejection fraction (EF). The left ventricular volume (LVV) is defined as the volume enclosed by the LV blood pool and the myocardial mass is equal to the volume of the myocardium, multiplied by the density of the myocardium:

(17) $$\text{Myo-Mass}=\text{Myo-Volume}\left({\text{cm}}^{3}\right)\times 1.06\left(\text{gram}/{\text{cm}}^{3}\right)$$

Stroke volume (SV) is defined as the volume ejected during systole and is equal to the difference between the end-diastolic volume (EDV) and the end-systolic volume (ESV):

(18) SV=EDV-ESV×100%

The ejection fraction (EF) is an important cardiac parameter quantifying the cardiac output and defined as the ratio of the SV to the EDV:

(19) $$EF=\frac{SV}{EDV}\times 100\%$$

## 3. Results

### 3.1. Image Segmentation Assessment

**Figure 8.**
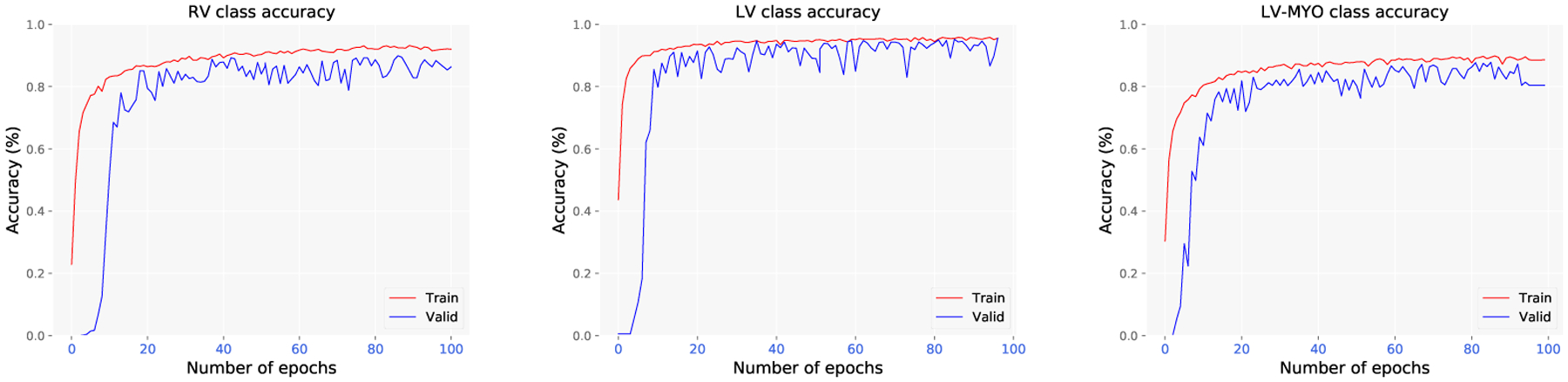
Representative accuracy curves showing the training and validation accuracy of three different classes (RV blood-pool, LV blood-pool, and LV-Myocardium).

We tested our C*q*SL model on varying proportions of labeled and unlabeled data available through the STACOM 2017 ACDC cine cardiac MRI dataset. Training and validation segmentation accuracies for three different classes (RV, LV, and LV-Myo) are shown in Figure 8 for 100 epochs. Note that the validation curves show similar trends as the training curves (Figure 8).

The C*q*SL experimental results were compared against a fully supervised U-Net model trained from scratch, as reported in Tables 1–3. Furthermore, to explore the effectiveness of each component in our model, we propose three different semi-supervised ablations, i.e., model **I**: only a GAN architecture (Figure 3c); model **II: I** + reconstruction (Figure 3c,d); model **III: II** + disentangler block (Figure 3a–d), which are also reported in Tables 1–3. The detailed comparison of our model can be seen in Table 4. The segmentation performance is evaluated both qualitatively and quantitatively. As shown in Tables 1–3, our proposed model significantly improves the segmentation performance of right ventricle (RV), left ventricle blood-pool (LV), and LV-Myocardium, respectively on varying proportions of annotated data in terms of the Dice and Jaccard indices, Hausdorff distance, precision and recall rates. Our C*q*SL model achieves a high dice score (± std. dev.) of 75.50±10.9% for the RV, 83.21±7.1% for the LV blood-pool and 77.65±9.3% for the LV-Myocardium even if we use only 1% labeled data.

**Figure 9.**
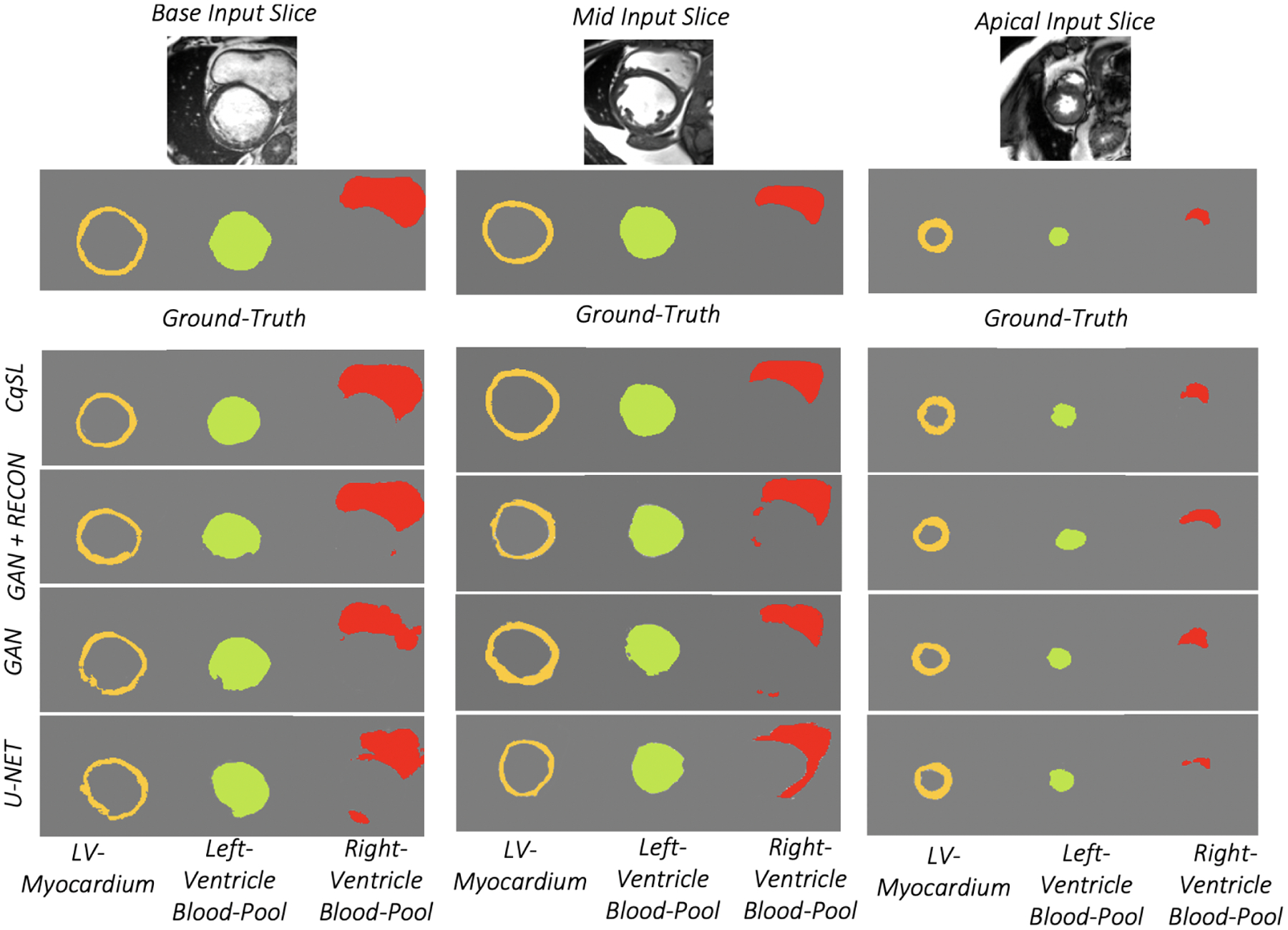
Representative results showing the comparison across several best performing networks, including C*q*SL for the semantic segmentation of full cardiac image dataset from the base to apex showing of RV blood-pool, LV blood-pool, and LV-Myocardium on 20% labeled data in red, green, and yellow respectively.

Figure 9 illustrates a qualitative segmentation output that compared C*q*SL and two others semi-supervised models, i.e., model **I**: only a GAN architecture (Figure 3c); model **II: I** + reconstruction (Figure 3c,d). For simplicity, this comparison is based on 20% unlabeled training data. As demonstrated, when only 20% of the training annotation is employed, U-Net fails completely to segment the cardiac structures from base to apex, particularly RV segmentation. As shown in the figure, the segmentation results improve with each consecutive addition of a distinct block. The GAN-only architecture performs badly, particularly during RV segmentation, whereas the addition of a reconstruction block improves performance. Finally, adding a disentangled block to the GAN and reconstruction block yielded the greatest results. Even the least performing version of our proposed CqSL model ${\;}^{4}\text{C}q\text{SL}$ achieves an overall accuracy superior to the U-Net, GAN-only, as well as GAN+REC model, confirming that the proposed model is able to effectively learn correct features that ensure correct segmentation.

**Figure 10.**
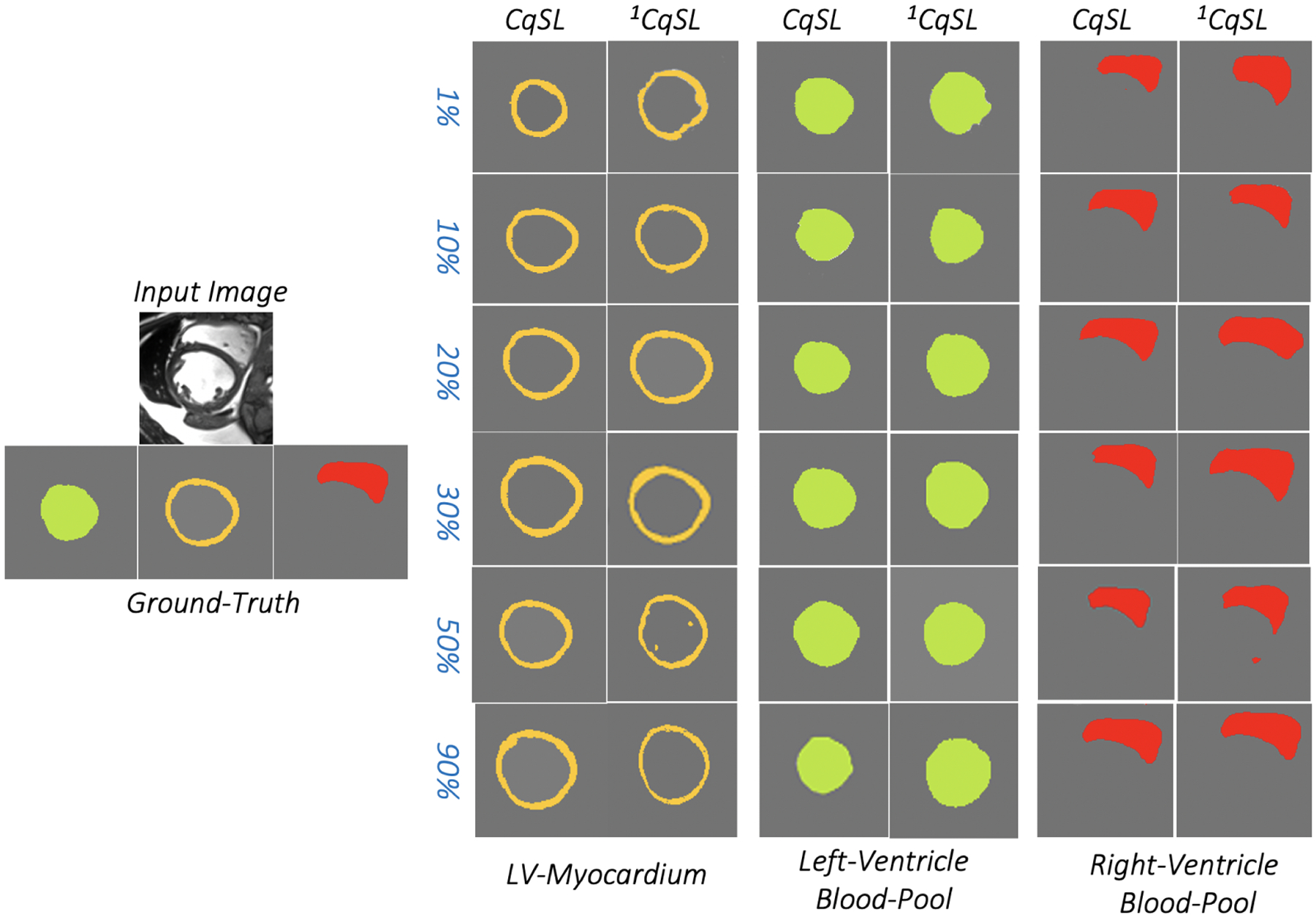
Representative results showing the semantic segmentation of RV, LV blood-pool, and LV-Myocardium on different proportion of labeled data in red, green, and yellow, respectively.

Figure 10 illustrates a qualitative segmentation output that compared C*q*SL and U-Net results with increasing proportion of unlabeled training data. For simplicity, we have shown two of our best performing models. As shown, when only 1% training annotation is used, U-Net completely fails to segment the cardiac structures. Under similar conditions, our model is still able to yield a high segmentation accuracy of LV, RV, and LV-Myocardium. When the amount of labeled data increases from 1% to 10%, the U-Net model still performs poorly, especially for RV segmentation. On the other hand, although the performance of our model improves significantly when utilizing more than 30% annotated data, its performance with even 1% labeled data is still satisfactory, comparable to that of semi-supervised models, and superior to U-Net’s performance under similar conditions.

We assessed the performance of our proposed C*q*SL cardiac image segmentation method against the segmentation results yielded by the well-established, fully supervised U-Net architecture [55] in light of its effectiveness across various medical image segmentation applications, as well as its extensive use as a baseline method for comparison by the participants of the ACDC cardiac image segmentation challenge. Furthermore, to explore the effectiveness of each component in our model, we experiment on three different semi-supervised ablations, i.e., model **I**: only a GAN architecture; model **II**: GAN + reconstruction; and model **III**: GAN + reconstruction + disentangler block (C*q*SL).

**Figure 11.**
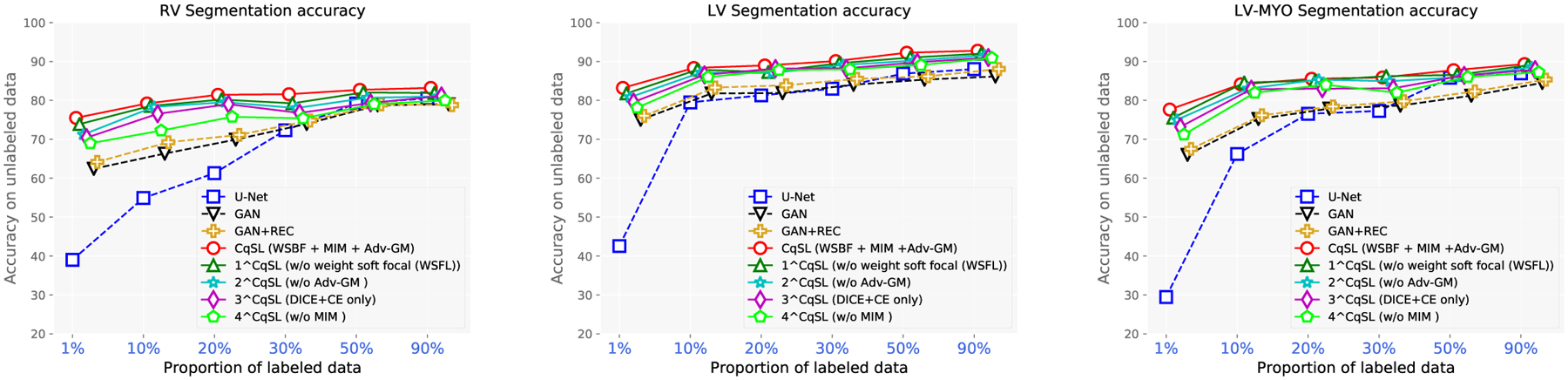
Consistent improvement in segmentation accuracy by the proposed C*q*SL model over baseline semi-supervised (variants of our C*q*SL model: ${\;}^{1}\text{C}q\text{SL},{\;}^{2}\text{C}q\text{SL},{\;}^{3}\text{C}q\text{SL}$, and ${\;}^{4}\text{C}q\text{SL}$) and fully supervised models in varying proportions of labeled training data.

**Figure 12.**
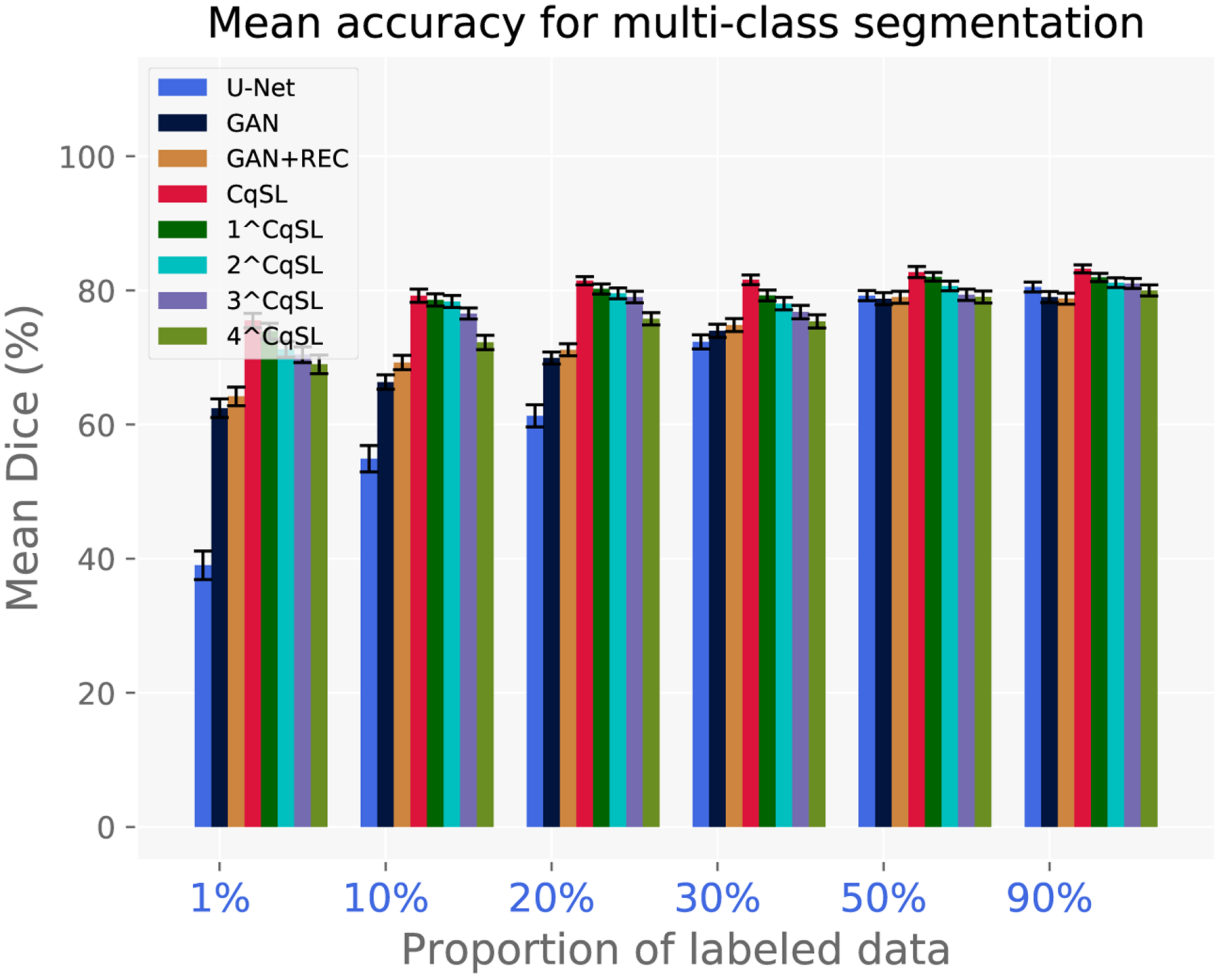
Evaluation on the robustness of C*q*SL in terms of mean accuracy over RV, LV, and LV-Myocardium segmentation tasks on varying amounts of labeled training samples. Note significant improvement in Dice score across all CqSL semi-supervised variants for as little as 1% unlabeled data.

**Figure 13.**
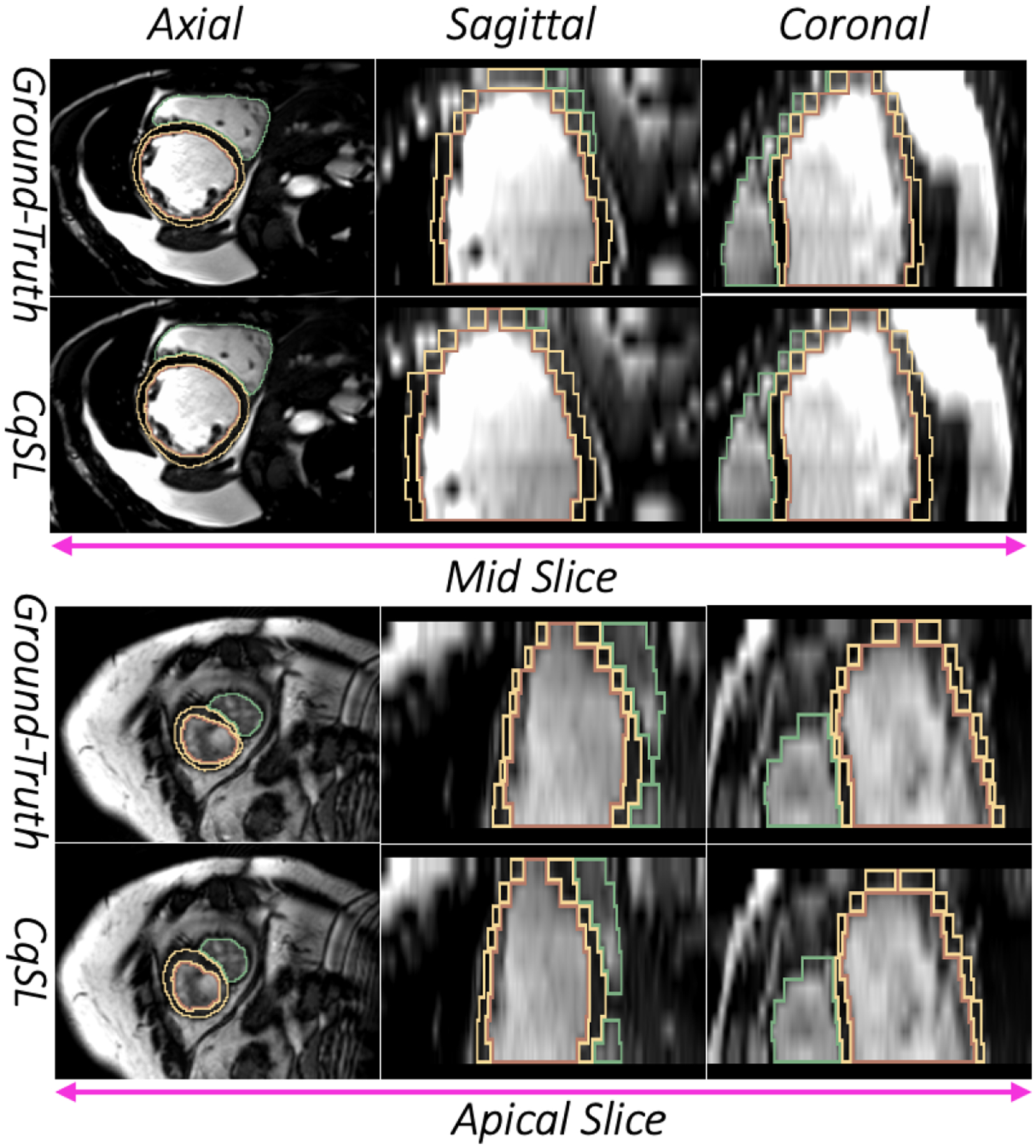
Representative segmentation contours of a complete cardiac cycle for the middle and apex slices showing RV and LV blood-pool, and LV-Myocardium in green, yellow, and brown, respectively, in three different view settings (axial, sagittal, and coronal).

As shown in Figure 11, the accuracy of our C*q*SL models remains high when using as much as 50–90% unlabeled data, which essentially implies excellent performance with as little as as 10% annotated data. Nevertheless, both U-Net and C*q*SL models perform similar to each other when the amount of annotated data increases above 90%. We plot the mean accuracy for all the models in Figure 12 and confirm that under low amounts of annotated data conditions, even as low as 1%, our proposed C*q*SL model and all four of its semi-supervised variants $\left({\;}^{1}\text{C}q\text{SL},{\;}^{2}\text{C}q\text{SL},{\;}^{3}\text{C}q\text{SL}\right.$, and $\left.{\;}^{4}\text{C}q\text{SL}\right)$ outperform GAN, GAN+REC, as well as U-Net models for LV, RV, and LV-Myocardium. The typical segmentation contours of complete cardiac image dataset for the mid and apical slices are shown in Figure 13.

### 3.2. Image Quality Assessment

**Figure 14.**
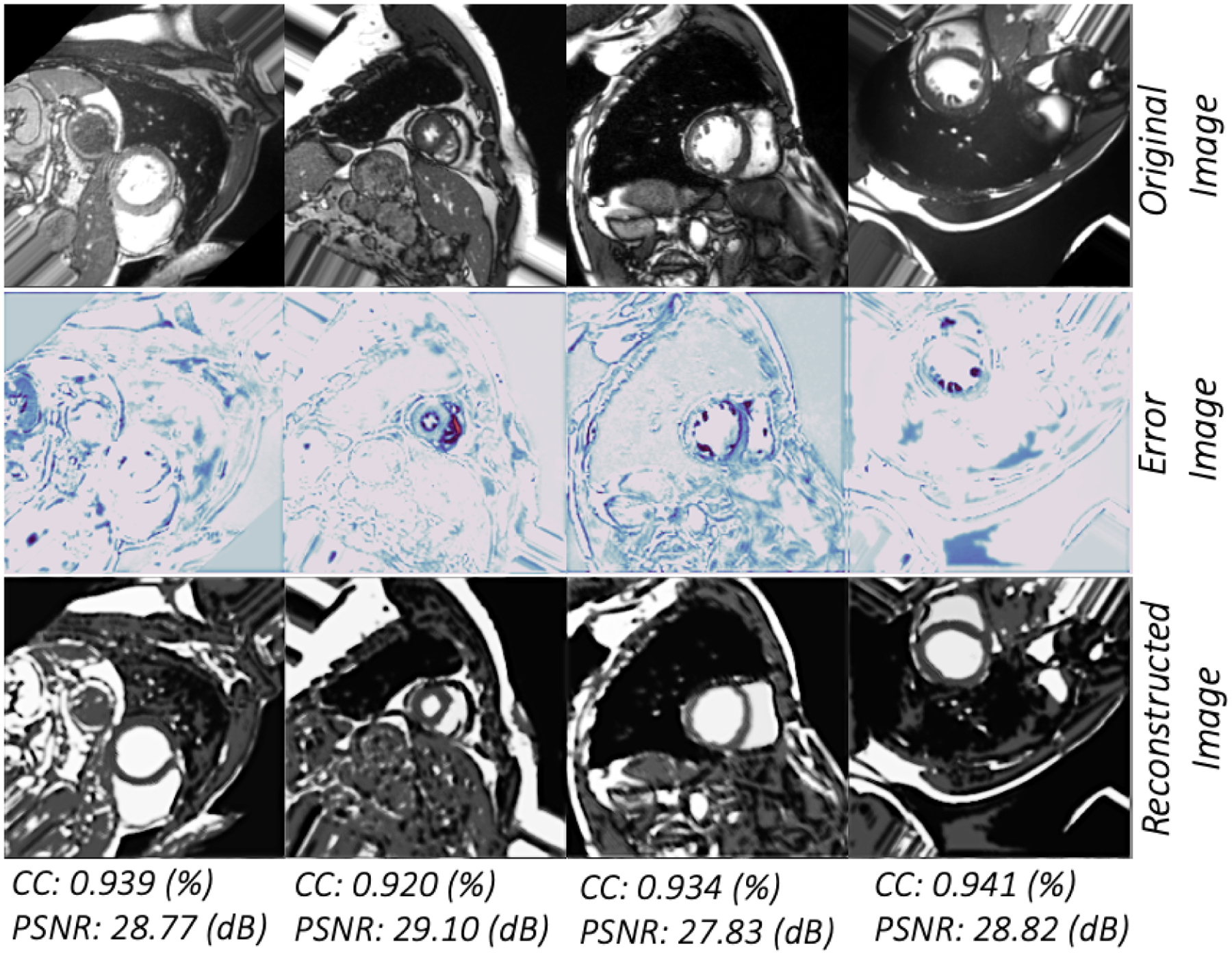
Qualitative comparison of the original and the reconstructed slices showing that the original images are well reconstructed by combining skeleton and sentiency information.The comparison is augmented by the computed correlation coefficients (CC) and peak signal-to-noise ratio (PSNR). The middle row illustrates the error images.

Figure 14 illustrates a qualitative comparison between the original image slice and the reconstructed slices generated from our proposed approach on the ACDC dataset at the original 5 mm slice thickness. The comparison is augmented by the computed correlation coefficients (CC) and peak signal-to-noise ratio (PSNR) shown below each figure. As illustrated in Figure 14, our approach preserves the fine structural details and realistic textures while remaining visually comparable to the ground truth image. Aside from qualitative improvements, the proposed method’s CC and PSNR values also prove that the synthesized image slices preserve the fine structural details.

**Figure 15.**
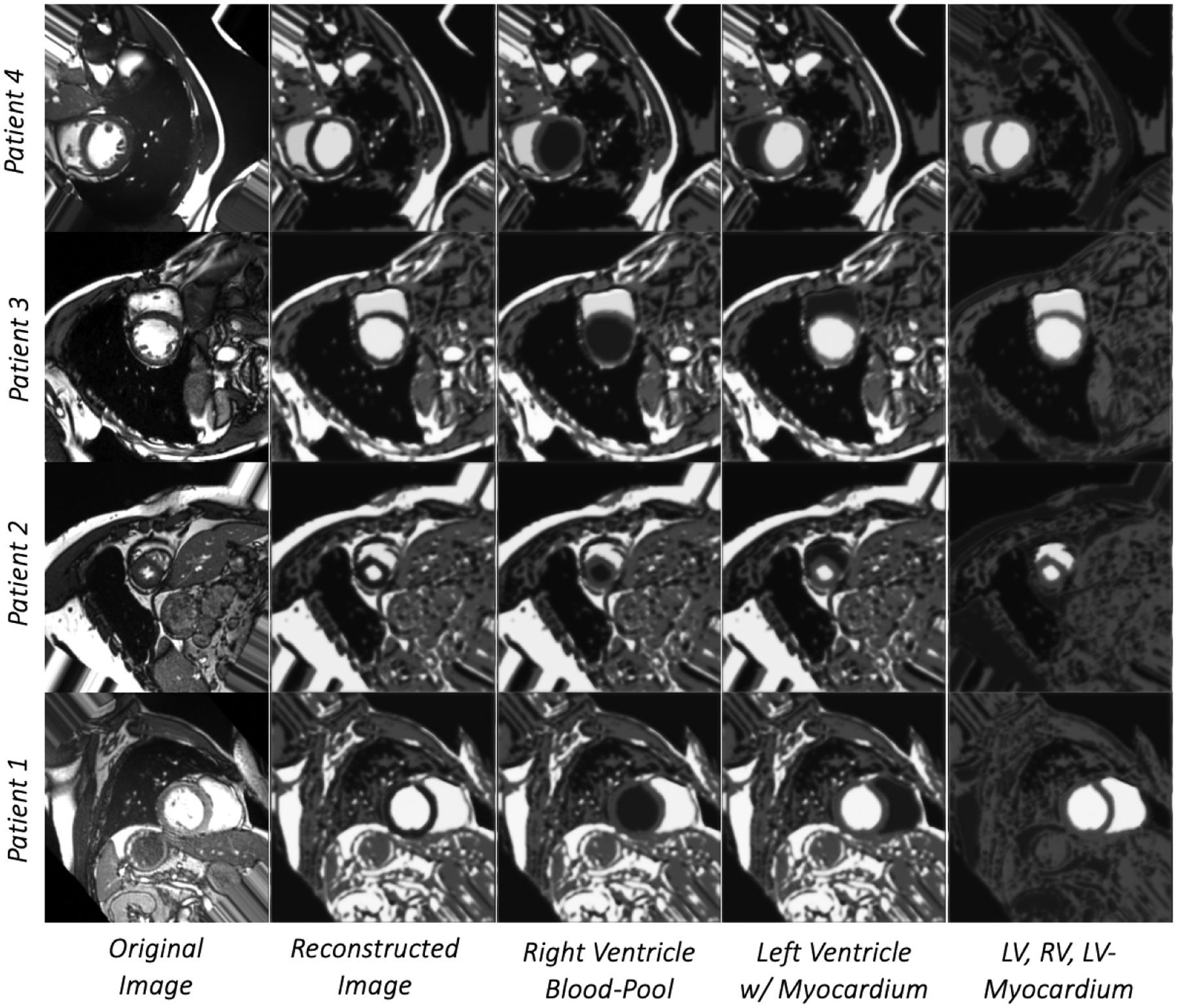
Reconstructions of a sample of input images when rearranging the spatial representation’s channels. Rearranging the channels results in reconstructing only left ventricle blood-pool or only right ventricle blood-pool only or all the ventricular structures.

Table 5 shows the quantitative results of the objective quality metrics of reconstruction, indicating that the use of feature-wise linear modulation to remove domain-invariant information from the disentangled latent code guides the synthesis of more texture information. Starting with the spatial factor, we change the content of the spatial channels in Figure 15 to see how the decoder has learned a correlation between the position of each channel and different signal intensities of the skeleton parts. The sentiency factor remains constant in all of these experiments. The first two columns show the original input and the reconstruction. The third row is created by the RV spatial channels and disregarding (zeroing) the MYO and LV channel. In the fourth image, we swap the RV channels with those of LV. Finally, the fifth column is produced by considering all LV, MYO and RV channels.

### 3.3. Clinical Parameter Estimation

The performance of our developed segmentation method was also reflected in the computed clinical indices. These clinical indices are computed using the Simpsons method and the agreement between the ground truth and the same parameters computed using the automated segmentation results is reported using correlation statistical analysis by mapping the predicted volumes of the testing set onto the ground truth volumes of the training set. As illustrated in Table 6 the agreement between our method’s prediction and ground truth is high, characterized by a Pearson’s correlation coefficient (rho) of 0.898 (p<0.01) for LV-EF, 0.723 for RV-EF (p<0.1) and 0.924(p<0.01) for Myo-mass. There was a slight over-estimation in the RV blood-pool segmentation also reflected in the clinical parameters estimation.

**Figure 16.**
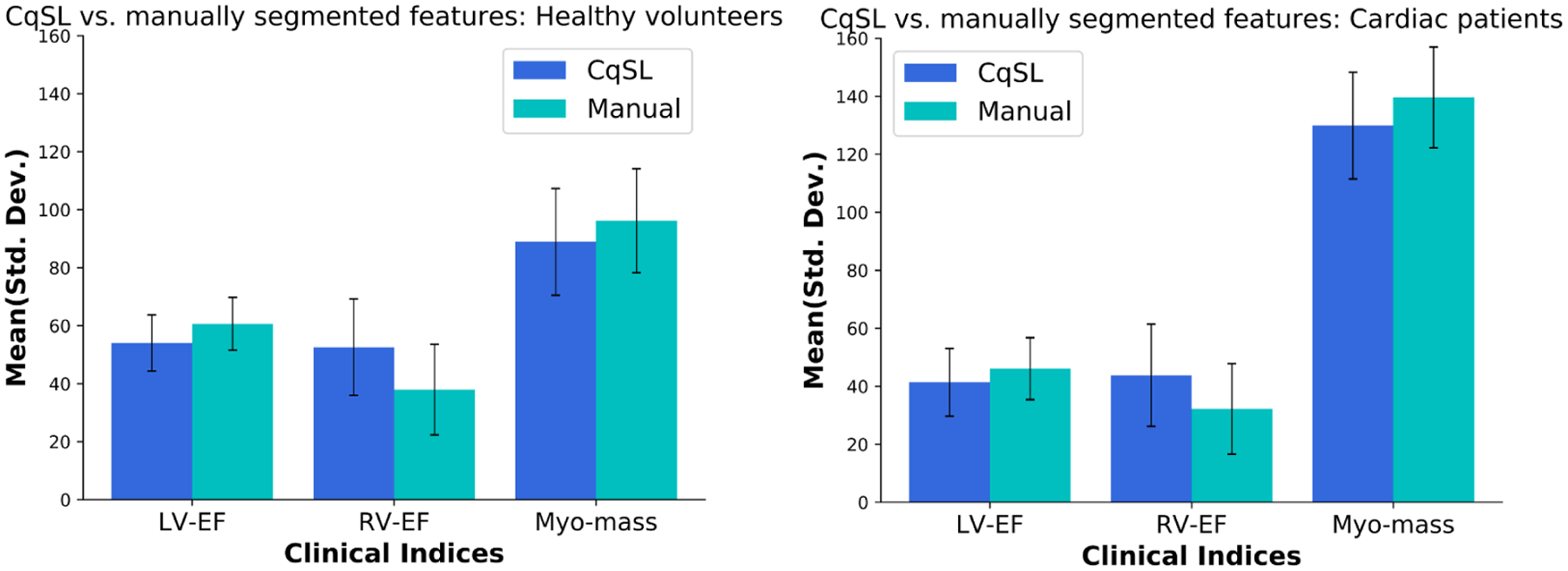
Graphical comparison showing no statistically significant differences between clinical parameters estimated using C*q*SL segmentation and same parameters estimated using the ground truth segmentation in terms of Mean (Std. Dev.) EF (mL/mL (%)) = ejection fraction, Myo-mass (in gm) = myocardial mass.

Figure 16 shows a graphical comparison between the clinical parameters estimated from the cardiac features segmented via C*q*SL and the same homologous parameters estimated from the ground truth manual segmentations for both healthy volunteers and patients featuring various cardiac conditions. As shown, the clinical parameters estimated using our automatically segmented features show no statistically significant difference from those estimated based on the ground truth, manually segmented features.

### 3.4. Ablation Studies

**Figure 17.**
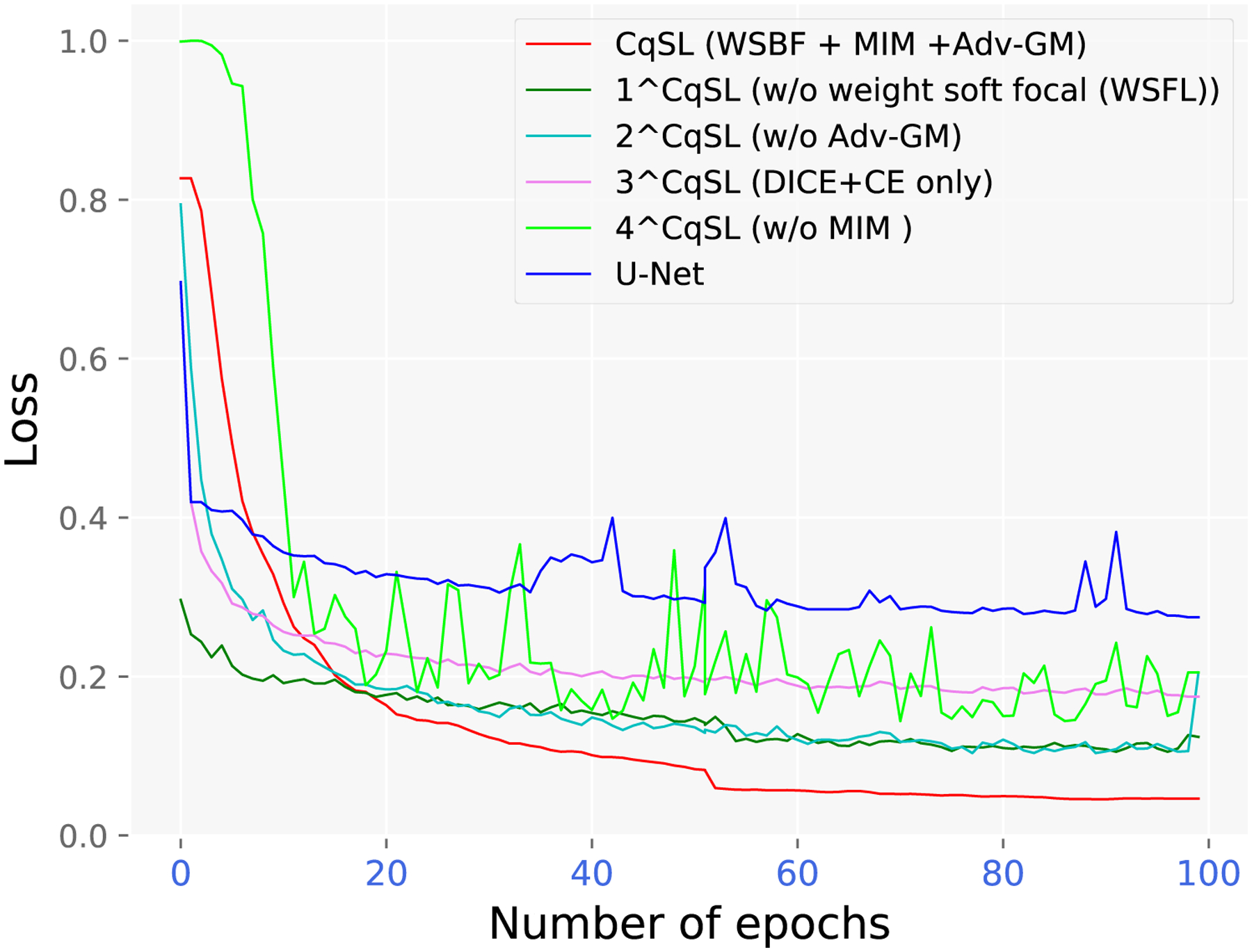
Empirical analysis showing the effect of different loss functions on the 2017 STACOM ACDC dataset. The significant reduction of total loss in C*q*SL (in red) suggests the best performing model with best learned features.

We perform an ablation study to investigate the effect of using different loss functions in our semi-supervised setting. We demonstrate the effect of different novel loss functions used in C*q*SL model: WSBF, MIM, and Adv-GM by assessing the model performance when each novel loss functions is removed. Figure 17 shows a graphical representation of the results achieved on the ACDC dataset. In Figure 10, we illustrate the qualitative results on the ACDC dataset to visualize the effect of using all of the loss components. We can observe that the best results are achieved when all of the loss components are used. Specifically, without MIM, the loss curve oscillates, while without WSBF, the output images deviate drastically from the ground truth. Both the quantitative and qualitative results show that the design of C*q*SL improves the preservation of the subject identity and enables more accurate segmentation of cardiac structures.

## 4. Conclusions and Future Work

In this paper, we propose a semi-supervised learning model (C*q*SL) that features multiple novel loss functions, including mutual information minimization (MIM), which minimizes the mutual information between the domain-invariant as well as domain-specific features. Empirically, we show that disentanglement with mutual information can improve the performance of the segmentation accuracy, while combined with an adversarial and a reconstruction block. Our novel use of total loss function enforces the network to capture both the spatial and intensity information. Our weighted soft focal loss can minimize the class imbalance problem by applying varying weights over different classes along with a modulating term. We apply the proposed model to cardiac image segmentation tasks with varying proportion of labeled data.

Our proposed C*q*SL model achieves 85% accuracy, significantly outperforming other baselines. We incrementally add each component, aiming to study their effectiveness on the final results: (model **I**: only a GAN architecture (Figure 3c); model **II**: GAN + reconstruction (Figure 3c,d); model **III**: GAN + reconstruction + disentangled block (Figure 3a–d).

In light of consistency, all four implemented C*q*SL variants are evaluated and compared to the baselines, but as shown in Tables 1–3, the first variant $\left({\;}^{1}CqS\right)$ performs best and hence it is deemed as the most suitable and recommended C*q*SL framework.

The experimental results reported in this manuscript show that the proposed *CqSL* framework outperforms semi-supervised learning with GANs [56] as well as fully supervised-type models when using as little as even 1% labeled data and display similar performance and comparable accuracy when employing more than 50% labeled data. Unlike these, we use adversarial-Geman–McClure (adv-GM) loss to force mask generation to be spatially aligned with the image. Furthermore, we discover that the semi-supervised segmentation approach of Hung et al. [18] obtains results slightly inferior to ours. Hung et al. reported that their adversarial model achieved a 80.63% accuracy when trained on 20% labeled data using the ACDC dataset, whereas our model achieved a 81.44% accuracy under similar training conditions.

Hence, the proposed method is the first to achieve significant performance for 4D cine cardiac MRI image segmentation with very minimal annotated data, specifically 1% of the training dataset. This is a key feature of the proposed work and hence a significant contribution to the medical (cardiac, in particular) image segmentation, as access to large amounts of expert-annotated ground truth imaging data is expensive in the medical field. Nevertheless, here we demonstrate that C*q*SL can still yield segmentation accuracy superior to other semi-supervised methods while requiring minimal annotated data for training.

## Figures and Tables

**Table 1.** Quantitative evaluation of RV blood pool segmentation results achieved using four semi-supervised variants of the proposed C*q*SL model in terms of mean Dice score (%) with std. dev., Jaccard index, Hausdorff distance (mm), precision (%) and recall (%) rate evaluated for varying proportions of labeled data on the ACDC dataset compared across several frameworks.

	Right Ventricle (RV)				
	Dice	Jaccard	HD	Prec.	Rec.
U-Net-90%	80.50 ± 8.45	72.03 ± 9.77	8.89 ± 8.45	90.09	94.35
U-Net-50%	79.21 ± 8.49	70.26 ± 10.69	8.90 ± 6.12	85.32	90.11
U-Net-30%	72.32 ± 10.60	66.10 ± 14.75	10.19 ± 7.43	79.50	83.45
U-Net-20%	61.29 ± 16.59	55.65 ± 18.90	12.88 ± 7.32	67.19	74.50
U-Net-10%	54.90 ± 19.66	46.89 ± 20.05	14.58 ± 9.03	60.55	63.02
U-Net-1.0%	39.02 ± 21.22	32.10 ± 22.22	15.90 ± 9.12	43.02	44.15
GAN-90%	79.0 ± 8.15	70.59 ± 10.89	9.55 ± 6.35	85.09	90.12
GAN-50%	78.76 ± 8.98	70.16 ± 11.18	9.88 ± 6.44	84.32	89.43
GAN-30%	73.97 ± 10.87	67.01 ± 13.04	10.23 ± 6.98	79.93	84.97
GAN-20%	69.92 ± 11.45	63.65 ± 16.88	11.66 ± 7.14	79.12	84.12
GAN-10%	66.33 ± 13.21	60.18 ± 19.23	11.99 ± 7.88	74.12	78.34
GAN-1.0%	62.43 ± 13.23	56.43 ± 22.12	13.43 ± 8.11	69.12	73.33
GAN+REC-90%	78.78 ± 8.11	71.13 ± 9.77	9.12 ± 6.46	86.09	90.23
GAN+REC-50%	78.98 ± 8.88	70.13 ± 11.13	9.78 ± 6.66	85.12	90.54
GAN+REC-30%	74.83 ± 10.67	68.67 ± 14.06	10.01 ± 6.98	80.12	85.32
GAN+REC-20%	71.14 ± 11.18	66.65 ± 16.44	11.34 ± 7.05	80.23	84.23
GAN+REC-10%	69.24 ± 13.78	63.23 ± 17.71	11.80 ± 7.23	75.13	79.12
GAN+REC-1.0%	64.19 ± 12.22	59.33 ± 21.01	12.91 ± 7.54	70.34	74.67
C*q*SL-90%	83.0 ± 6.33	77.77 ± 11.66	8.1 ± 6.00	90.78	95.12
C*q*SL-50%	82.72 ± 8.29	76.15 ± 11.0	8.21 ± 6.04	88.44	94.26
C*q*SL-30%	81.59 ± 7.20	73.27 ± 12.14	8.28 ± 6.10	85.19	92.62
C*q*SL-20%	81.44 ± 6.12	75.33 ± 11.52	8.56 ± 6.11	83.14	93.79
C*q*SL-10%	79.21 ± 9.76	71.45 ± 12.91	9.82 ± 6.78	82.40	90.93
C*q*SL-1.0%	75.50 ± 10.87	70.55 ± 12.58	9.87 ± 6.72	80.55	83.68
^1^C*q*SL-90%	81.88 ± 6.0	74.31 ± 11.65	8.5 ± 6.15	90.12	91.97
^1^C*q*SL-50%	82.03 ± 6.45	75.22 ± 11.24	8.49 ± 6.10	88.11	93.44
^1^C*q*SL-30%	79.25 ± 8.11	73.16 ± 8.14	8.77 ± 6.22	83.62	92.05
^1^C*q*SL-20%	80.21 ± 7.54	73.19 ± 11.04	9.01 ± 6.34	83.69	91.05
^1^C*q*SL-10%	78.58 ± 9.22	71.12 ± 11.25	9.48 ± 6.57	82.21	91.01
^1^C*q*SL-1.0%	73.90 ± 11.88	68.58 ± 13.89	9.85 ± 6.71	79.54	84.54
^2^C*q*SL-90%	81.03 ± 7.11	74.37 ± 11.48	8.74 ± 6.25	88.39	92.28
^2^C*q*SL-50%	80.65 ± 7.26	73.36 ± 12.06	8.54 ± 6.23	86.78	93.05
^2^C*q*SL-30%	78.02 ± 9.36	72.66 ± 10.55	9.35 ± 6.65	82.88	91.96
^2^C*q*SL-20%	79.55 ± 8.10	73.0 ± 11.54	9.65 ± 6.63	83.02	89.15
^2^C*q*SL-10%	78.33 ± 8.96	68.54 ± 12.89	9.77 ± 6.34	80.56	91.55
^2^C*q*SL-1.0%	71.21 ± 11.76	63.45 ± 15.91	11.82 ± 7.12	76.40	81.93
^3^C*q*SL-90%	81.13 ± 7.33	73.04 ± 12.11	8.93 ± 6.33	86.02	90.17
^3^C*q*SL-50%	79.34 ± 8.56	71.23 ± 12.87	9.05 ± 6.66	84.34	91.24
^3^C*q*SL-30%	76.77 ± 10.11	72.04 ± 11.26	9.66 ± 6.73	82.0	90.88
^3^C*q*SL-20%	79.01 ± 8.58	71.89 ± 12.88	9.52 ± 6.46	81.66	87.56
^3^C*q*SL-10%	76.55 ± 8.25	68.55 ± 13.23	10.12 ± 6.89	81.02	88.72
^3^C*q*SL-1.0%	70.41 ± 11.86	64.77 ± 15.70	12.11 ± 7.23	74.44	80.21
^4^C*q*SL-90%	79.83 ± 8.23	70.33 ± 12.66	9.25 ± 6.34	84.54	90.02
^4^C*q*SL-50%	79.02 ± 8.88	72.68 ± 12.26	9.36 ± 6.23	85.20	90.22
^4^C*q*SL-30%	75.38 ± 9.75	70.49 ± 12.0	9.52 ± 6.54	80.33	88.59
^4^C*q*SL-20%	75.77 ± 9.05	69.88 ± 13.22	10.19 ± 6.77	81.02	88.78
^4^C*q*SL-10%	72.24 ± 10.65	66.70 ± 13.56	10.55 ± 6.75	79.79	85.47
^4^C*q*SL-1.0%	68.97 ± 13.90	63.19 ± 16.50	12.88 ± 7.43	72.13	77.59

**Table 2.** Quantitative evaluation of LV blood pool segmentation results achieved using four semi-supervised variants of the proposed C*q*SL model in terms of mean Dice score (%) with std. dev., Jaccard index, Hausdorff distance (mm), precision (%) and recall (%) rates evaluated for varying proportions of labeled data on the ACDC dataset compared across several frameworks.

	Left Ventricle (LV)				
	Dice	Jaccard	HD	Prec.	Rec.
U-Net-90%	88.03 ± 6.81	85.09 ± 6.98	5.16 ± 5.92	97.88	98.79
U-Net-50%	86.88 ± 6.09	84.67 ± 5.36	5.29 ± 6.20	97.01	98.19
U-Net-30%	82.98 ± 8.66	80.10 ± 8.19	6.89 ± 6.75	89.66	91.05
U-Net-20%	81.29 ± 8.91	79.78 ± 9.02	8.22 ± 8.23	87.50	89.77
U-Net-10%	79.49 ± 9.56	71.29 ± 11.26	9.56 ± 9.82	83.33	86.14
U-Net-1.0%	42.56 ± 19.76	37.02 ± 21.45	14.35 ± 10.12	45.53	46.17
GAN-90%	86.15 ± 6.45	81.23 ± 8.01	5.53 ± 5.08	90.57	92.87
GAN-50%	85.34 ± 7.03	81.26 ± 8.12	5.91 ± 6.03	88.34	89.43
GAN-30%	84.03 ± 8.16	80.22 ± 9.11	6.89 ± 7.03	87.23	88.87
GAN-20%	81.90 ± 8.59	79.12 ± 10.82	7.12 ± 7.33	86.19	88.12
GAN-10%	81.78 ± 8.16	76.67 ± 14.13	8.02 ± 7.54	83.15	87.43
GAN-1.0%	75.02 ± 12.32	70.22 ± 15.12	10.89 ± 9.12	80.22	83.12
GAN+REC-90%	88.06 ± 6.11	81.94 ± 8.12	5.73 ± 5.22	91.19	93.35
GAN+REC-50%	86.19 ± 6.89	81.02 ± 8.23	5.76 ± 5.43	90.54	91.65
GAN+REC-30%	85.53 ± 7.36	80.34 ± 9.12	6.78 ± 6.34	89.76	90.34
GAN+REC-20%	83.89 ± 8.19	79.34 ± 10.22	6.88 ± 7.05	87.19	89.53
GAN+REC-10%	83.29 ± 7.16	77.56 ± 13.05	7.58 ± 8.33	85.55	89.02
GAN+REC-1.0%	76.02 ± 11.22	71.32 ± 14.22	10.04 ± 9.12	80.12	84.43
C*q*SL-90%	92.77 ± 4.98	85.67 ± 7.31	4.53± 4.98	96.12	99.75
C*q*SL-50%	92.25 ± 5.12	83.98 ± 7.98	5.23 ± 5.03	95.91	97.95
C*q*SL-30%	90.10 ± 5.89	82.91 ± 8.12	5.93 ± 5.23	93.50	93.79
C*q*SL-20%	88.98 ± 6.33	81.26 ± 8.78	6.21 ± 5.04	90.14	92.90
C*q*SL-10%	88.33 ± 6.39	79.92 ± 9.21	6.17 ± 6.44	89.35	92.95
C*q*SL-1.0%	83.21 ± 7.12	77.94 ± 10.51	7.0 ± 5.98	86.96	91.36
^1^C*q*SL-90%	92.21 ± 5.13	83.66 ± 7.45	4.88 ± 3.21	95.03	97.33
^1^C*q*SL-50%	91.0 ± 5.55	81.61 ± 8.05	5.16 ± 4.09	94.12	96.13
^1^C*q*SL-30%	89.56 ± 5.97	81.23 ± 7.89	5.89 ± 6.98	92.22	92.80
^1^C*q*SL-20%	87.28 ± 6.91	80.32 ± 8.12	6.55 ± 5.23	89.89	91.0
^1^C*q*SL-10%	87.89 ± 6.44	79.15 ± 9.30	6.05 ± 5.33	89.03	92.55
^1^C*q*SL-1.0%	81.78 ± 7.22	75.36 ± 9.20	7.88 ± 5.44	84.55	89.17
^2^C*q*SL-90%	91.45 ± 5.86	83.31 ± 7.23	4.90 ± 4.90	95.13	96.73
^2^C*q*SL-50%	90.22 ± 5.12	80.78 ± 8.34	5.54 ± 4.55	93.02	96.04
^2^C*q*SL-30%	89.11 ± 5.89	81.14 ± 8.10	5.88 ± 5.11	91.14	92.89
^2^C*q*SL-20%	87.02 ± 6.98	81.12 ± 8.77	6.74 ± 5.28	89.11	90.58
^2^C*q*SL-10%	87.15 ± 6.93	79.02 ± 8.87	6.44 ± 4.87	88.53	92.47
^2^C*q*SL-1.0%	80.80 ± 8.12	75.06 ± 10.04	8.01 ± 6.12	85.54	90.20
^3^C*q*SL-90%	91.03 ± 5.57	82.44 ± 7.87	5.32 ± 4.77	95.31	95.55
^3^C*q*SL-50%	89.79 ± 5.02	79.15 ± 8.04	5.12 ± 5.12	93.44	95.18
^3^C*q*SL-30%	89.24 ± 6.15	81.02 ± 7.95	5.71 ± 5.18	92.26	91.11
^3^C*q*SL-20%	88.19 ± 5.53	80.52 ± 8.12	6.80 ± 5.05	88.78	89.10
^3^C*q*SL-10%	86.56 ± 6.15	79.55 ± 8.45	6.56 ± 6.54	87.98	92.01
^3^C*q*SL-1.0%	79.58 ± 9.25	73.20 ± 10.87	8.64 ± 7.01	85.77	91.05
^4^C*q*SL-90%	90.55 ± 5.88	80.19 ± 8.25	6.55 ± 6.12	93.12	95.55
^4^C*q*SL-50%	89.10 ± 6.15	79.01 ± 8.77	5.54 ± 5.88	92.11	93.22
^4^C*q*SL-30%	88.01 ± 6.43	79.89 ± 8.00	5.86 ± 6.43	91.54	91.02
^4^C*q*SL-20%	87.78 ± 5.53	80.13 ± 7.72	6.91 ± 5.16	88.17	90.56
^4^C*q*SL-10%	86.0 ± 6.39	80.10 ± 8.90	6.92 ± 5.12	85.67	93.34
^4^C*q*SL-1.0%	78.13 ± 8.66	74.19 ± 11.20	9.56 ± 8.05	84.66	89.10

**Table 3.** Quantitative evaluation of LV-Myocardium segmentation results achieved using four semi-supervised variants of the proposed C*q*SL model in terms of mean Dice score (%) with std. dev., Jaccard index, Hausdorff distance (mm), precision (%) and recall (%) evaluated for varying proportions of labeled data on the ACDC dataset compared to segmentation across several frameworks.

	LV-Myocardium (LV-Myo)				
	Dice	Jaccard	HD	Prec.	Rec.
U-Net-90%	86.93 ± 5.56	84.50 ± 5.20	4.97 ± 3.76	92.32	96.54
U-Net-50%	85.82 ± 6.32	82.25 ± 7.66	5.16 ± 5.77	90.19	95.66
U-Net-30%	77.29 ± 9.19	75.49 ± 7.90	6.56 ± 5.65	87.11	89.56
U-Net-20%	76.56 ± 9.16	71.78 ± 16.20	7.69 ± 5.45	83.57	88.34
U-Net-10%	66.23 ± 15.90	60.63 ± 19.87	10.10 ± 8.55	59.34	62.08
U-Net-1.0%	29.47 ± 20.29	25.39 ± 22.50	13.95 ± 9.12	32.25	34.54
GAN-90%	84.50 ± 6.14	79.03 ± 9.17	5.89 ± 4.23	88.12	89.14
GAN-50%	81.21 ± 7.49	74.12 ± 11.77	5.45 ± 5.14	85.55	88.01
GAN-30%	78.67 ± 9.61	75.88 ± 12.75	5.19 ± 6.15	84.33	86.10
GAN-20%	77.88 ± 9.89	72.45 ± 15.91	6.01 ± 7.65	83.32	85.12
GAN-10%	75.23 ± 11.19	70.33 ± 17.19	7.87 ± 8.55	76.44	81.33
GAN-1.0%	66.02 ± 20.10	62.55 ± 20.87	12.67 ± 9.72	71.43	76.23
GAN+REC-90%	85.34 ± 6.42	77.44 ± 12.13	5.34 ± 4.37	88.44	90.33
GAN+REC-50%	82.33 ± 7.49	75.16 ± 13.16	5.81 ± 4.73	87.32	89.10
GAN+REC-30%	79.77 ± 9.21	74.10 ± 14.77	5.91 ± 5.12	86.76	88.34
GAN+REC-20%	78.43 ± 9.11	73.32 ± 15.11	6.12 ± 6.14	84.12	87.43
GAN+REC-10%	76.18 ± 11.18	72.21 ± 15.80	7.23 ± 7.34	79.43	83.53
GAN+REC-1.0%	67.52 ± 18.12	64.22 ± 19.33	12.12 ± 9.34	72.43	78.44
C*q*SL-90%	89.33 ± 5.11	82.03 ± 7.33	5.20 ± 5.11	93.98	96.01
C*q*SL-50%	87.77 ± 6.19	79.12 ± 9.0	5.88 ± 5.43	93.33	93.17
C*q*SL-30%	85.89 ± 7.07	77.72 ± 11.92	6.23 ± 6.14	91.20	92.25
C*q*SL-20%	85.55 ± 7.22	76.95 ± 12.9	6.85 ± 7.04	90.01	91.09
C*q*SL-10%	84.14 ± 7.64	72.76 ± 13.01	7.07 ± 8.01	88.84	90.88
C*q*SL-1.0%	77.65 ± 9.26	74.20 ± 11.87	10.88 ± 8.45	83.22	88.10
^1^C*q*SL-90%	88.98 ± 6.01	81.78 ± 7.63	6.11 ± 6.10	94.13	95.33
^1^C*q*SL-50%	86.55 ± 6.22	78.31 ± 9.46	5.74 ± 5.34	93.41	94.11
^1^C*q*SL-30%	86.23 ± 7.62	77.43 ± 11.89	6.43 ± 6.29	91.88	91.0
^1^C*q*SL-20%	85.10 ± 6.98	76.09 ± 12.77	6.80 ± 6.25	88.87	91.09
^1^C*q*SL-10%	84.56 ± 8.01	72.11 ± 13.54	8.13 ± 7.03	89.73	90.16
^1^C*q*SL-1.0%	75.54 ± 9.89	73.01 ± 11.56	10.05 ± 8.43	80.89	85.44
^2^C*q*SL-90%	88.44 ± 6.43	81.03 ± 7.89	6.65 ± 5.24	92.0	95.32
^2^C*q*SL-50%	86.01 ± 6.69	79.28 ± 10.02	5.65 ± 5.27	93.19	92.66
^2^C*q*SL-30%	84.93 ± 8.01	78.52 ± 11.61	6.88 ± 5.86	90.42	93.53
^2^C*q*SL-20%	85.33 ± 5.73	77.11 ± 11.59	6.32 ± 7.32	89.82	92.38
^2^C*q*SL-10%	83.02 ± 8.33	71.67 ± 14.04	8.71 ± 8.10	87.77	91.45
^2^C*q*SL-1.0%	75.0 ± 10.10	72.55 ± 11.18	10.20 ± 8.88	81.01	86.56
^3^C*q*SL-90%	87.33 ± 7.22	80.73 ± 8.10	6.43 ± 5.50	92.31	94.52
^3^C*q*SL-50%	86.43 ± 6.32	78.56 ± 10.22	5.76 ± 5.40	91.34	92.11
^3^C*q*SL-30%	83.10 ± 8.66	78.15 ± 10.78	5.92 ± 6.11	88.82	91.63
^3^C*q*SL-20%	83.00 ± 6.02	75.44 ± 13.10	6.65 ± 7.63	90.31	92.11
^3^C*q*SL-10%	82.88 ± 9.01	72.00 ± 14.66	7.98 ± 8.34	86.11	90.87
^3^C*q*SL-1.0%	73.19 ± 11.56	70.04 ± 12.93	10.78 ± 8.54	77.50	83.39
^4^C*q*SL-90%	87.44 ± 7.71	81.24 ± 7.45	6.12 ± 5.11	91.32	92.65
^4^C*q*SL-50%	86.01 ± 6.81	76.12 ± 10.64	6.01 ± 6.12	89.32	91.88
^4^C*q*SL-30%	81.98 ± 10.01	76.65 ± 11.44	5.32 ± 5.44	87.11	92.33
^4^C*q*SL-20%	84.01 ± 7.44	75.15 ± 13.19	6.72 ± 6.41	88.43	91.66
^4^C*q*SL-10%	81.97 ± 10.66	73.43 ± 13.78	6.69 ± 6.87	84.77	86.32
^4^C*q*SL-1.0%	71.21 ± 11.76	69.25 ± 13.16	11.82 ± 9.23	75.40	82.56

**Table 4.** Our proposed C*q*SL model achieves 84.9% accuracy, significantly outperforming other baselines. We incrementally add each component, aiming to study their effectiveness on the final results; (model **I**: only a GAN architecture (Figure 3c); model **II**: GAN + reconstruction (Figure 3c,d); model III: GAN + reconstruction + disentangled block (Figure 3a–d). ↑ denotes higher the value better the result; ↓ denotes lower the value better the result.

	Average				
Models	Dice ↑	Jaccard ↑	HD ↓	Prec. ↑	Rec. ↑
**Model I:** GAN	76.56 ± 9.97	71.74 ± 14.54	8.26 ± 7.37	82.87 ± 7.66	85.78 ± 6.34
**Model II:** GAN + REC	77.82 ± 9.87	73.10 ± 13.92	8.11 ± 6.74	83.84 ± 7.12	87.06 ± 5.65
**Model III:** GAN + REC + DISENTANGLE (C*q*SL)	**84.92** ± **6.55**	**77.85** ± **11.06**	**7.20** ± **6.06**	**87.76** ± **5.45**	**89.56** ± 5.04

**Table 5.** Image reconstruction assessment: correlation coefficient (CC) and PSNR comparison between reconstructed and input images based on 288 test sets.

	Reconstruction Quality	
	CC (%)	PSNR (dB)
	n = 288	n = 288
**Model II:** GAN + REC	0.912	27.32
**Model III:** GAN + REC + DISENTANGLE (Proposed)	0.934	28.89

**Table 6.** The correlation between the C*q*SL-predicted and ground truth clinical indices is significantly higher than the correlation between the U-Net-predicted and same ground truth clinical indices (** (*p* < 0.01), * (*p* < 0.1)).

	Clinical Indices of Healthy Volunteers	
	UNet	C*q*SL
LV EF	0.487	0.898 **
RV EF	0.371	0.723 *
Myo mass	0.427	0.924 **

## References

[1] Bhowmik A; Gumhold S; Rother C; Brachmann E Reinforced feature points: Optimizing feature detection and description for a high-level task. In Proceedings of the IEEE/CVF Conference on Computer Vision and Pattern Recognition, Seattle, WA, USA, 13–19 June 2020; pp. 4948–4957.

[2] Li S; Wang Z; Liu Z; Tan C; Lin H; Wu D; Chen Z; Zheng J; Li SZ Efficient Multi-order Gated Aggregation Network. arXiv 2022, arXiv:2211.03295

[3] Ruan J; Xiang S; Xie M; Liu T; Fu Y MALUNet: A Multi-Attention and Light-weight UNet for Skin Lesion Segmentation. arXiv 2022, arXiv:2211.01784

[4] Tack J; Yu S; Jeong J; Kim M; Hwang SJ; Shin J Consistency regularization for adversarial robustness. In Proceedings of the AAAI Conference on Artificial Intelligence, Virtually, 22 February–1 March 2022; Volume 36, pp. 8414–8422.

[5] Sajjadi M; Javanmardi M; Tasdizen T Regularization with stochastic transformations and perturbations for deep semi-supervised learning. In Proceedings of the Advances in Neural Information Processing Systems, Barcelona, Spain, 5–10 December 2016; pp. 1163–1171.

[6] Elakkiya R; Subramaniyaswamy V; Vijayakumar V; Mahanti A Cervical cancer diagnostics healthcare system using hybrid object detection adversarial networks. IEEE J. Biomed. Health Inform 2021, 26, 1464–1471. 10.1109/JBHI.2021.3094311 34214045

[7] Hasan SMK; Linte C STAMP: A Self-training Student-Teacher Augmentation-Driven Meta Pseudo-Labeling Framework for 3D Cardiac MRI Image Segmentation. In Proceedings of the Annual Conference on Medical Image Understanding and Analysis, Cambridge, UK, 27–29 July 2022; Springer: Berlin/Heidelberg, Germany, 2022; pp. 371–386. 10.1007/978-3-031-12053-4_28 PMC10134897 37126464

[8] Sohn K; Berthelot D; Li CL; Zhang Z; Carlini N; Cubuk ED; Kurakin A; Zhang H; Raffel C Fixmatch: Simplifying semi-supervised learning with consistency and confidence. arXiv 2020, arXiv:2001.07685

[9] Xie Q; Luong MT; Hovy E; Le QV Self-training with noisy student improves imagenet classification. In Proceedings of the IEEE/CVF Conference on Computer Vision and Pattern Recognition, Seattle, WA, USA, 13–19 June 2020; pp. 10687–10698.

[10] Bai W; Oktay O; Sinclair M; Suzuki H; Rajchl M; Tarroni G; Glocker B; King A; Matthews PM; Rueckert D Semi-supervised learning for network-based cardiac MR image segmentation. In Proceedings of the International Conference on Medical Image Computing and Computer-Assisted Intervention, Quebec City, QC, Canada, 10–14 September 2017; Springer: Berlin/Heidelberg, Germany, 2017; pp. 253–260.

[11] Donahue J; Krähenbühl P; Darrell T Adversarial feature learning. arXiv 2016, arXiv:1605.09782

[12] Saito K; Kim D; Sclaroff S; Darrell T; Saenko K Semi-supervised domain adaptation via minimax entropy. In Proceedings of the IEEE International Conference on Computer Vision, Seoul, Republic of Korea, 27 October–2 November 2019; pp. 8050–8058.

[13] Gomes HM; Grzenda M; Mello R; Read J; Le Nguyen MH; Bifet A A survey on semi-supervised learning for delayed partially labelled data streams. ACM Comput. Surv. (CSUR) 2022, 55, 1–42.

[14] Hasan SMK; Linte CA A Multi-Task Cross-Task Learning Architecture for Ad Hoc Uncertainty Estimation in 3D Cardiac MRI Image Segmentation. In Proceedings of the 2021 Computing in Cardiology (CinC), Brno, Czech Republic, 13–15 September 2021; Volume 48, pp. 1–4. 10.23919/cinc53138.2021.9662869 PMC9137435 35647207

[15] Chan ER; Lin CZ; Chan MA; Nagano K; Pan B; De Mello S; Gallo O; Guibas LJ; Tremblay J; Khamis S; Efficient geometry-aware 3D generative adversarial networks. In Proceedings of the IEEE/CVF Conference on Computer Vision and Pattern Recognition, New Orleans, LA, USA, 19–24 June 2022; pp. 16123–16133.

[16] Souly N; Spampinato C; Shah M Semi supervised semantic segmentation using generative adversarial network. In Proceedings of the IEEE International Conference on Computer Vision, Venice, Italy, 22–29 October 2017; pp. 5688–5696.

[17] Chen C; Dou Q; Chen H; Heng PA Semantic-aware generative adversarial nets for unsupervised domain adaptation in chest X-ray segmentation. In Proceedings of the International Workshop on Machine Learning in Medical Imaging, Granada, Spain, 16 September 2018; Springer: Berlin/Heidelberg, Germany, 2018; pp. 143–151.

[18] Hung WC; Tsai YH; Liou YT; Lin YY; Yang MH Adversarial learning for semi-supervised semantic segmentation. arXiv 2018, arXiv:1802.07934

[19] Zhang Y; Yang L; Chen J; Fredericksen M; Hughes DP; Chen DZ Deep adversarial networks for biomedical image segmentation utilizing unannotated images. In Proceedings of the International Conference on Medical Image Computing and Computer-Assisted Intervention, Quebec City, QC, Canada, 10–14 September 2017; Springer: Berlin/Heidelberg, Germany, 2017; pp. 408–416.

[20] Chartsias A; Joyce T; Dharmakumar R; Tsaftaris SA Adversarial image synthesis for unpaired multi-modal cardiac data. In Proceedings of the International Workshop on Simulation and Synthesis in Medical Imaging, Quebec City, QC, Canada, 10 September 2017; Springer: Berlin/Heidelberg, Germany, 2017; pp. 3–13.

[21] Hjelm RD; Fedorov A; Lavoie-Marchildon S; Grewal K; Bachman P; Trischler A; Bengio Y Learning deep representations by mutual information estimation and maximization. arXiv 2018, arXiv:1808.06670

[22] Wang YC; Wang CY; Lai SH Disentangled Representation with Dual-stage Feature Learning for Face Anti-spoofing. In Proceedings of the IEEE/CVF Winter Conference on Applications of Computer Vision, Waikoloa, HI, USA, 3–8 January 2022; pp. 1955–1964.

[23] Siddharth N; Paige B; Van de Meent JW; Desmaison A; Goodman N; Kohli P; Wood F; Torr P Learning disentangled representations with semi-supervised deep generative models. In Proceedings of the Advances in Neural Information Processing Systems, Long Beach, CA, USA, 4–9 December 2017; pp. 5925–5935.

[24] Higgins I; Matthey L; Pal A; Burgess C; Glorot X; Botvinick M; Mohamed S; Lerchner A beta-vae: Learning Basic Visual Concepts with a Constrained Variational Framework. 2016. Available online: https://openreview.net/forum?id=Sy2fzU9gl (accessed on 2 October 2022).

[25] Bengio Y; Courville A; Vincent P Representation learning: A review and new perspectives. IEEE Trans. Pattern Anal. Mach. Intell 2013, 35, 1798–1828. 23787338 10.1109/TPAMI.2013.50

[26] Lipton ZC The mythos of model interpretability. Queue 2018, 16, 31–57.

[27] Schölkopf B; Janzing D; Peters J; Sgouritsa E; Zhang K; Mooij J On causal and anticausal learning. arXiv 2012, arXiv:1206.6471

[28] Isola P; Zhu JY; Zhou T; Efros AA Image-to-image translation with conditional adversarial networks. In Proceedings of the IEEE Conference on Computer Vision and Pattern Recognition, Honolulu, HI, USA, 21–26 July 2017; pp. 1125–1134.

[29] Zhu JY; Park T; Isola P; Efros AA Unpaired image-to-image translation using cycle-consistent adversarial networks. In Proceedings of the IEEE International Conference on Computer Vision, Venice, Italy, 22–29 October 2017; pp. 2223–2232.

[30] Huang X; Liu MY; Belongie S; Kautz J Multimodal unsupervised image-to-image translation. In Proceedings of the European Conference on Computer Vision (ECCV), Munich, Germany, 8–14 September 2018; pp. 172–189.

[31] Shen K; Jones RM; Kumar A; Xie SM; HaoChen JZ; Ma T; Liang P Connect, not collapse: Explaining contrastive learning for unsupervised domain adaptation. In Proceedings of the International Conference on Machine Learning, PMLR, Baltimore, MD, USA, 17–23 July 2022; pp. 19847–19878.

[32] Gatys LA; Ecker AS; Bethge M Image style transfer using convolutional neural networks. In Proceedings of the IEEE Conference on Computer Vision and Pattern Recognition, Las Vegas, NV, USA, 37-30 June 2016; pp. 2414–2423.

[33] Liu AH; Liu YC; Yeh YY; Wang YCF A unified feature disentangler for multi-domain image translation and manipulation. In Proceedings of the Advances in Neural Information Processing Systems, Montreal, QC, Canada, 3–8 December 2018; pp. 2590–2599.

[34] Tzeng E; Hoffman J; Saenko K; Darrell T Adversarial discriminative domain adaptation. In Proceedings of the IEEE Conference on Computer Vision and Pattern Recognition, Honolulu, HI, USA, 21–26 July 2017; pp. 7167–7176.

[35] Ulyanov D; Vedaldi A; Lempitsky V Improved texture networks: Maximizing quality and diversity in feed-forward stylization and texture synthesis. In Proceedings of the IEEE Conference on Computer Vision and Pattern Recognition, Honolulu, HI, USA, 21–26 July 2017; pp. 6924–6932.

[36] Dumoulin V; Shlens J; Kudlur M A learned representation for artistic style. arXiv 2016, arXiv:1610.07629

[37] Huang X; Belongie S Arbitrary style transfer in real-time with adaptive instance normalization. In Proceedings of the IEEE International Conference on Computer Vision, Venice, Italy, 22–29 October 2017; pp. 1501–1510.

[38] Perez E; Strub F; De Vries H; Dumoulin V; Courville A Film: Visual reasoning with a general conditioning layer. In Proceedings of the Thirty-Second AAAI Conference on Artificial Intelligence, New Orleans, LA, USA, 2–7 February 2018.

[39] Park T; Liu MY; Wang TC; Zhu JY Semantic image synthesis with spatially-adaptive normalization. In Proceedings of the IEEE Conference on Computer Vision and Pattern Recognition, Long Beach, CA, USA, 15–20 June 2019; pp. 2337–2346.

[40] Marino J Predictive coding, variational autoencoders, and biological connections. Neural Comput. 2022, 34, 1–44. 10.1162/neco_a_01458 34758480

[41] Kim H; Mnih A Disentangling by factorising. arXiv 2018, arXiv:1802.05983

[42] Zhou Z; Rahman Siddiquee MM; Tajbakhsh N; Liang J Unet++: A nested u-net architecture for medical image segmentation. In Deep Learning in Medical Image Analysis and Multimodal Learning for Clinical Decision Support; Springer: Berlin/Heidelberg, Germany, 2018; pp. 3–11. 10.1007/978-3-030-00889-5_1 PMC7329239 32613207

[43] Tian R; Mao Y; Zhang R Learning VAE-LDA models with rounded reparameterization trick. In Proceedings of the 2020 Conference on Empirical Methods in Natural Language Processing (EMNLP), Virtually, 16–20 November 2020; pp. 1315–1325.

[44] Chen X; Duan Y; Houthooft R; Schulman J; Sutskever I; Abbeel P Infogan: Interpretable representation learning by information maximizing generative adversarial nets. In Proceedings of the Advances in Neural Information Processing Systems, Barcelona, Spain, 5–10 December 2016; pp. 2172–2180.

[45] Peng X; Huang Z; Sun X; Saenko K Domain agnostic learning with disentangled representations. arXiv 2019, arXiv:1904.12347

[46] Mao X; Li Q; Xie H; Lau RY; Wang Z; Paul Smolley S Least squares generative adversarial networks. In Proceedings of the IEEE International Conference on Computer Vision, Venice, Italy, 22–29 October 2017; pp. 2794–2802.

[47] Ganan S; McClure D Bayesian Image Analysis: An Application to Single Photon Emission Tomography; American Statistical Association: Washington, DC, USA, 1985; pp. 12–18.

[48] Bernard O; Lalande A; Zotti C; Cervenansky F; Yang X; Heng PA; Cetin I; Lekadir K; Camara O; Ballester MAG; Deep learning techniques for automatic MRI cardiac multi-structures segmentation and diagnosis: Is the problem solved? IEEE Trans. Med. Imaging 2018, 37, 2514–2525. 29994302 10.1109/TMI.2018.2837502

[49] Chartsias A; Joyce T; Papanastasiou G; Semple S; Williams M; Newby DE; Dharmakumar R; Tsaftaris SA Disentangled representation learning in cardiac image analysis. Med. Image Anal 2019, 58, 101535. 31351230 10.1016/j.media.2019.101535 PMC6815716

[50] Ioffe S; Szegedy C Batch normalization: Accelerating deep network training by reducing internal covariate shift. In Proceedings of the International Conference on Machine Learning, PMLR, Lille, France, 7–9 July 2015; pp. 448–456.

[51] Maas AL; Hannun AY; Ng AY; Rectifier nonlinearities improve neural network acoustic models. In Proceedings of the International Conference on Machine Learning, Atlanta, GA, USA, 16–21 June 2013; Volume 30, p. 3.

[52] Radford A; Metz L; Chintala S Unsupervised representation learning with deep convolutional generative adversarial networks. arXiv 2015, arXiv:1511.06434

[53] Liu H; Brock A; Simonyan K; Le QV Evolving Normalization-Activation Layers. arXiv 2020, arXiv:2004.02967

[54] Frangi AF; Niessen WJ; Viergever MA Three-dimensional modeling for functional analysis of cardiac images, a review. IEEE Trans. Med. Imaging 2001, 20, 2–5. 11293688 10.1109/42.906421

[55] Ronneberger O; Fischer P; Brox T U-net: Convolutional networks for biomedical image segmentation. In Proceedings of the International Conference on Medical Image Computing and Computer-Assisted Intervention, Munich, Germany, 5–9 October 2015; Springer: Berlin/Heidelberg, Germany, 2015; pp. 234–241.

[56] Luc P; Couprie C; Chintala S; Verbeek J Semantic segmentation using adversarial networks. arXiv 2016, arXiv:1611.08408

